# Recent advances in surface-mounted metal–organic framework thin film coatings for biomaterials and medical applications: a review

**DOI:** 10.1186/s40824-023-00454-y

**Published:** 2023-11-10

**Authors:** Mohammad Mehdi Sabzehmeidani, Mahmood Kazemzad

**Affiliations:** 1 Department of Energy, Materials and Energy Research Center, Karaj, Iran; 2 Department of Chemical Engineering, University of Science and Technology of Mazandaran, Behshahr, Iran

**Keywords:** Surface modification, MOF Coatings, Thin film, Biocompatibility, Anti-corrosion

## Abstract

Coatings of metal–organic frameworks (MOFs) have potential applications in surface modification for medical implants, tissue engineering, and drug delivery systems. Therefore, developing an applicable method for surface-mounted MOF engineering to fabricate protective coating for implant tissue engineering is a crucial issue. Besides, the coating process was desgined for drug infusion and effect opposing chemical and mechanical resistance. In the present review, we discuss the techniques of MOF coatings for medical application in both in vitro and in vivo in various systems such as in situ growth of MOFs, dip coating of MOFs, spin coating of MOFs, Layer-by-layer methods, spray coating of MOFs, gas phase deposition of MOFs, electrochemical deposition of MOFs. The current study investigates the modification in the implant surface to change the properties of the alloy surface by MOF to improve properties such as reduction of the biofilm adhesion, prevention of infection, improvement of drugs and ions rate release, and corrosion resistance. MOF coatings on the surface of alloys can be considered as an opportunity or a restriction. The presence of MOF coatings in the outer layer of alloys would significantly demonstrate the biological, chemical and mechanical effects. Additionally, the impact of MOF properties and specific interactions with the surface of alloys on the anti-microbial resistance, anti-corrosion, and self-healing of MOF coatings are reported. Thus, the importance of multifunctional methods to improve the adhesion of alloy surfaces, microbial and corrosion resistance and prospects are summarized.

**Graphical Abstract:**

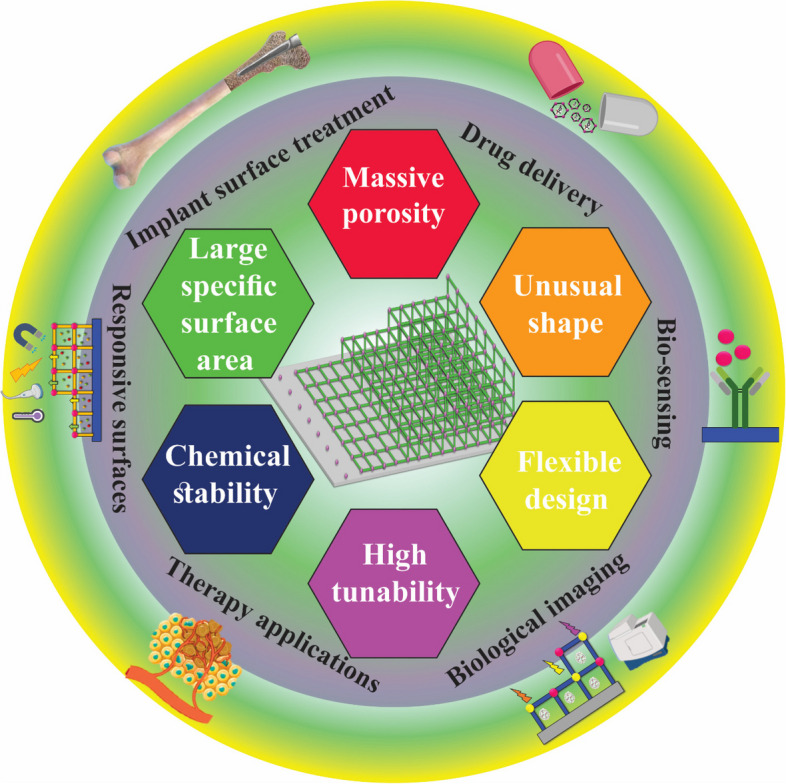

## Introduction

The high diversity of topological networks and attractive properties of metal–organic frameworks (MOFs) led to the development of their design and synthesis. MOFs, also recognized as porous polymers, are essentially composed of two main segments: a metal ion and an organic unit called a linker that typically includes di-, tri-, or tetradentate ligands [1, 2]. The porous hybrid solids-derived tubes (1D), layers (2D), and even frameworks (3D) were called MOFs [3, 4]. Various transition metal ions lead to diverse coordination numbers and geometries like linear, square planar, tetrahedral, square-pyramidal, trigonal–bipyramidal, and octahedral shapes [5]. Especially in the field of medicine, MOF materials are attracting much attention due to their unusual structures and properties, such as massive porosity and easy tunability of their pore size and shape from microporous to mesoporous scale [6]. The high internal porosity of MOFs provides spaces for enclosing different materials such as large amounts of drugs and biological molecules. Bio-MOFs typically would use natural organic ligands such as amino acids, proteins, peptides, nucleobases, porphyrins, and saccharides as bridges. The biological ligands have various coordination paths to metal centers that lead to the different structures of bio-MOFs [7]. In addition, other natural small molecules can also be applied as biological ligands to coordinate with metals to fabricate bio-MOFs for medical and surgical applications [8, 9]. The hybrid organic–inorganic MOFs allows the appropriate and optimal fabrication of molecular building blocks possessing favorable functionality and porosity can be selected for a system suitable for medical and surgical applications. The ability and passion to prepare porous MOFs is emerged from their various applicability for bio-sensing [10], drug delivery [11], biological imaging [12], biocatalysts [13], disease diagnosis [14], implants [15], and therapy treatment [16] compared with traditional materials. In addition, researchers are currently exploring high multifunctional platforms to satisfy the growing demand for medical care, diagnostic care, and preventive healthcare. The preparation of thin MOF coatings as promising approaches to biomaterials is the surface-mounted strategy, which involves the direct fabrication of MOFs on the substrate surface. This method allows control of the MOF thickness and crystal orientation, as well as the integration of functional groups into the MOF structure.

The chemical composition and outstanding structure of MOFs include a variety of advantages such as the high porosity morphology, the regular micro and mesopores shape, the great specific surface area, flexible design ability, and simple diffusion of reagents via pores [17, 18]. There are several drawbacks in some samples of MOFs, like relatively low yields and poor reproducibility [19]. The poor chemical stability is one of the main drawbacks of MOFs which depends largely on the metal node and the strength of the chemical bond between the metal node and the bonder. In general, typical MOF substances are unstable in water and are also gradually degraded by water molecules due to the interference of coordination bonds [20]. Zr-based MOFs are one of the best chemically stable MOFs in organic solvents and water under an extensive pH range as well as high thermal stability (near 375 °C) [19]. MOF materials also have their benefits and drawbacks for specific applications. The solvothermal and hydrothermal techniques have typically achieved the best results in crystallinity and morphological properties compared to the other preparation methods. In the solvothermal process, the complete removal of all organic solvents is very difficult and also causes a risk to biomedical applications in vivo. But in the hydrothermal method, water is used, which is the friendliest solvent [21]. The multidentate organic linkers also bonded generally via carboxylic or heterocyclic nitrogen moieties. Therefore, the study of coordination between the metal ions and biological ligands such as amino acids, peptides, and proteins can give us more insight into the flexibility and collapse of pores [22, 23]. For medical treatment, there are diverse limitations of drugs after encapsulating in the frameworks of MOF structures, such as poor solubility, blood instability, and systemic toxicity [8]. These challenges potentially restrict the empirical usage of MOF in biomedical applications. As regards, MOF thin film coatings are of interest due to their wide range of applications in the medical and surgical field. The application of MOF in the medical field requires a complete and extensive analysis of cellular biocompatibility and nano-safety. Among various MOFs, a group of isostructural MOF-74 s have been known principally due to their high surface area and active metal sites that are candidate for medical applications. For instance, Mg-MOF-74 for CO_2_ adsorption [24], Fe-MOF-74 for drug delivery of ibuprofen anions [25], Mg-MOF-74 for drug delivery of combined ibuprofen and curcumin [26], the mixing Mg-MOF-74 with Zn-MOF-74 for drug delivery of curcumin [27] that were studied.

**Fig. 1:**
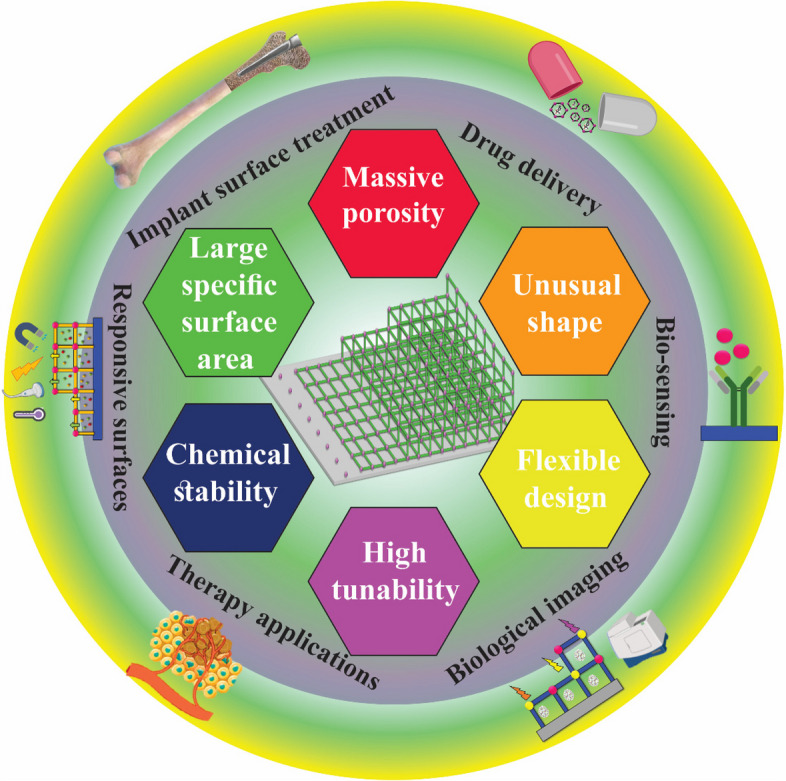
Schematic illustration of the characteristics and performance of MOF coatings, their application of biomaterials, and medical application

The present review mainly discusses the growth and medical usages of MOF films on substrate surfaces. We intend to fill this gap by examining the effects of various MOF coating for different medical uses ranging from drug delivery systems to surface coating of medical implants. This paper portrays insights concerning the flexible idea of coating MOFs for implant surface treatment, responsive surface, therapy, drug delivery, biological imaging, and bio-sensing (Fig. 1). The present paper gives a point-by-point audit of the various properties of MOF coating, including anti-corrosion and anti-microbial properties of MOF coating. This paper provides a brief review of multiple techniques of MOF coating on different substrates. In particular, it summarizes work focusing on peer growth and applying MOF coatings on substrate surfaces. Moreover, the future challenges and outlook of MOF coating are also discussed. This review may interest researchers attempting to fabricate other MOF coatings and those involved in expanding the medical coating applications of MOFs.

## Biomedical surface

**Fig. 2:**
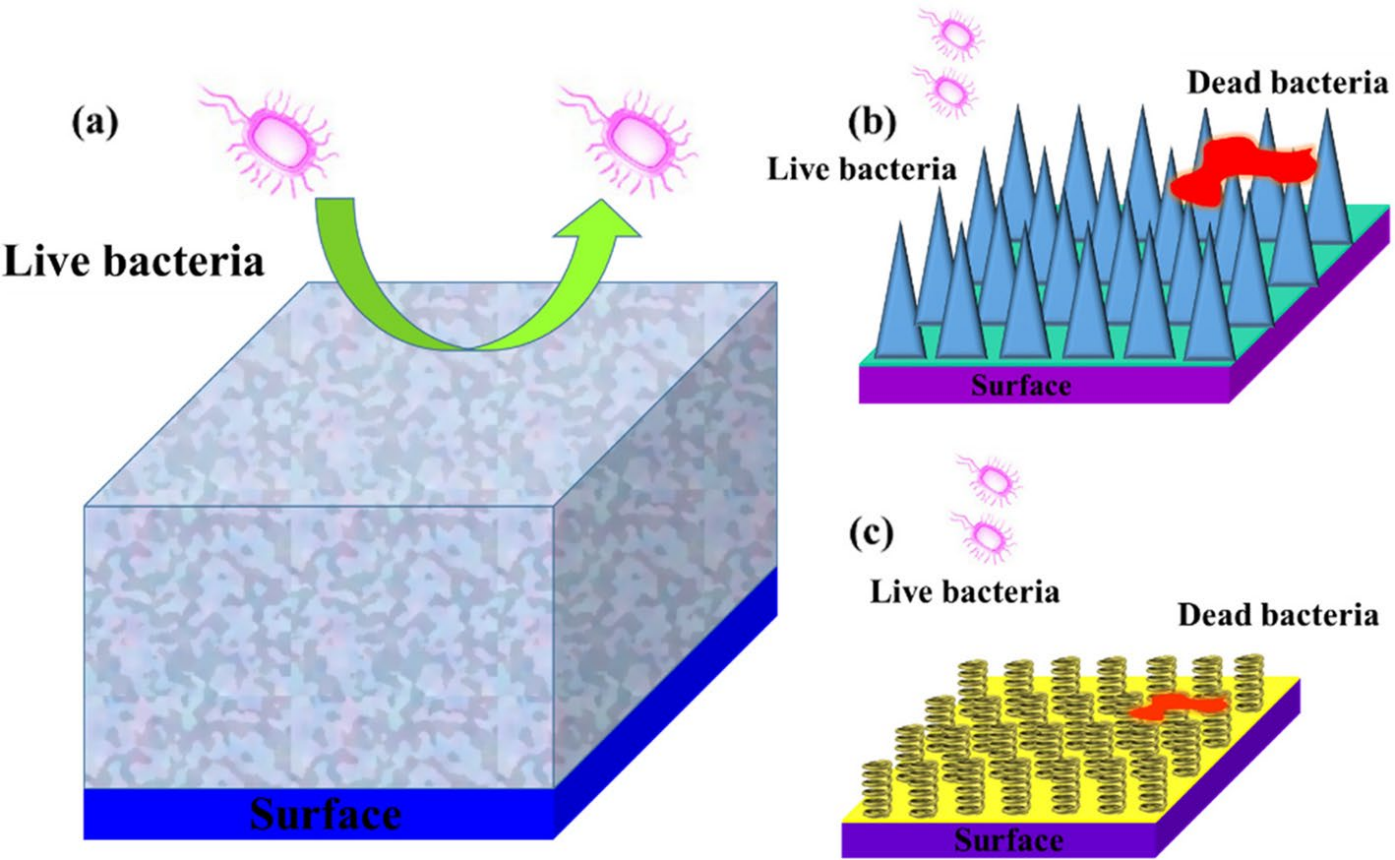
Schematic illustration anti-adhesive activity of the hydrogel coating (**a**), and the bacterial cell damage upon contact with nano-pillars aligned on the surface (**b**), the anti-microbial peptide coated surface with bactericidal activity upon contact (**c**)

Biomedical surfaces play an important and influencing role in various medical applications, such as implantable devices and tissue engineering scaffolds. These surfaces are designed to interact with biological systems, and the characteristics of the substrate and coating have a significant effect on their performance [28]. They play an essential role in ensuring biocompatibility, promoting tissue integrity, preventing infection and increasing their performance. The design and properties of these surfaces directly affect their biocompatibility, which refers to a material's ability to interact with living tissues without causing adverse reactions. A well-designed biomedical surface can significantly improve patient outcomes and the overall success of medical procedures [29, 30]. The properties of both substrates and coatings on biomedical surfaces straightly affect their biocompatibility, functionality, and overall performance. By understanding these features and using their application to develop innovative medical devices, improving patient care and advancing the field of healthcare is necessary [31]. The substrate is the basic component of biomedical surfaces and its properties affect the performance of the surface. One of the essential specifications of the biocompatible substrate is that it does not cause immune responses or toxicity in the body. Mechanical strength is another important factor that is very important in the substrate, as it must withstand physiological forces and stress without compromising its integrity. In addition, the surface topography of the substrate affects cell adhesion, proliferation and differentiation. Also, the roughness or smoothness of the substrate can strengthen or hinder these cellular processes. Another key factor is porosity, particularly in tissue engineering applications, where it allows tissue ingrowth and nutrient exchange. But some of these properties can be improved or modified by applying coating [32, 33]. Coatings are often applied to enhance substrate performance for biomedical surface applications. Biocompatibility and strong adhesion of the coating to the substrate is very important and ensures that the coating does not cause adverse reactions in the body and ensures strong adhesion to the substrate to prevent peeling. Coatings can also be designed to have antimicrobial properties and reduce the risk of infection. In addition, coatings can act as reservoirs for controlled drug release, enabling localized treatment and improved therapeutic outcomes [34, 35]. Surface modification is a common strategy for preparing new chemical, mechanical, and geometrical properties of the material. The adhesion of bacteria to the surface starts a process that leads to the formation of a biofilm by bacterial colonization and, eventually infection [36]. The current methods for disinfection modifications mostly include anti-adhesive polymer coatings, but MOF coatings are highly regarded [37–39] (Fig. 2a-c). In general, the surface modification of MOFs is widely applied for medical applications [40–42]. MOF coatings have been considered as an interesting biomedical surface and due to their unique properties in the field of biomedicine. They can be used as thin films on various substrates including medical implants to improve their performance and biocompatibility. MOF coatings have several unique properties in biomedical applications. They can be used as drug delivery systems, where the porous structure of MOFs can be used to entrap and release therapeutic agents in a controlled manner [43]. The large surface area of MOFs also allows effective adsorption of biomolecules such as proteins and enzymes, which can improve the biocompatibility and performance of implants. MOF coatings can be designed to have special properties such as anti-bacterial or anti-fouling capabilities that can help prevent infection and biofouling on biomedical surfaces. They can also be engineered to have stimuli-responsive properties, where the release of drugs or other molecules can be triggered by external stimuli, such as changes in pH or temperature [44]. The surface is coated by MOFs crystalline porous structure including a hybrid array of metal ions or clusters mounted to organic ligands. In general, there are several advantages of a MOF thin film compared to a metal oxide, such as porosity, selectivity, flexibility, surface area, tunability of coordination, synthesis, and processing. Besides, among the weak points of MOF thin films compared to other materials, we can mention techniques such as stability and low diffusion rate, complex and specialized processing, low mechanical strength, and relatively expensive cost. The ability to modify surface properties with MOFs to achieve improved corrosion resistance and biocompatibility while maintaining substrate material properties is a special advantage of surface-mounted MOF approaches.

## Challenges of bioactive and biocompatible coating

Bioactive and biocompatible coatings are a vital factor in various medical and biotechnological applications, such as implantable medical devices, drug delivery systems, and tissue engineering scaffolds. Due to their unique properties, these coatings can interact favorably with biological systems, leading to improved system integrity and minimizing adverse reactions. There are several technical challenges impeding the clinical applications of implant alloys. One of the critical challenges for the coating implant approach is maintaining the balance of obtaining efficient bactericidal ion concentrations without damaging the host tissue [45]. Furthermore, different architectural metal implants can be manufactured using a variation of accessible manufacturing technology [46]. Overall, there are several challenges, such as stability, biocompatibility, and controlled release of drugs, adhesion, and durability, cost-effectiveness associated with coating implants with MOFs. Overcoming challenges requires extensive research, collaboration between multidisciplinary teams, and the development of innovative techniques for MOF synthesis, characterization, and coating processes. Among the metallic implants, Ti-based alloys, Stainless steel, Co-based, and biodegradable Mg-based alloys are also highly available bone implant substances. The application of implants depends on various requirements the healing of the fractured bones, rectifying deformities, the improvement of the function of other parts of the human body, and the replacement of the damaged part of the anatomy [47]. For Ti alloys, extensive studies are yet to be carried out due to extremely challenging alloy processing through the melting casting route [48]. In addition, the low rate of destruction of traditional implants makes them unsuitable for temporary implant applications [49]. For Mg alloys, the challenge is that susceptibility to localized corrosion attack is when corrosion products cover their surface [50, 51]. The severe limitations of implant alloys lie in their inappropriate mechanical properties that lead to the stress-shielding severe problem, and the non-degradability of these materials. Sometimes these materials need a second surgery for implant elimination which leads to the release of toxic ions through the corrosion process [52]. The development of coatings with controlled development factor release rate is considered to simplify continuous bone conduction of Mg-based implants [53]. Therefore, the improvement and expansion of next-generation implant biomaterials is an urgent necessity. To develop bioactive and biocompatible coatings, there are several challenges, such as ensuring biocompatibility, stability in the long term, adhesion, exfoliation, controlled release, multiple functions, and biodegradability and absorption. Addressing these challenges requires interdisciplinary collaboration and advances in materials science, surface modification methods, and a deep understanding of biological interactions.

## Advantages and disadvantages of MOF coating

In general, the well-known MOFs are in powder form which are suitable for catalysts and sensor applications, but their usage as surface coatings is also of great interest. Then, thin or thick film depositions of MOFs have been explored due to their excellent surface area and abundant active sites due to their several applications of film MOFs in optical, medical, electronic, and energy devices [54–56]. It is noted as one of the efficient methods to design and fabricate MOF-coated implants using functional groups to make synergistic properties of alloy substrate and MOF coatings. It is believed that the unique MOF coating can significantly change the reactivity of the alloy surface [57]. Among the various thin film deposition techniques of MOFs, the liquid phase stepwise growth method makes it possible to prepare uniform and highly crystalline MOFs called surface-mounted MOFs (SURMOFs). This method is based on the consecutive immersion of functionalized substrate into the reagent solutions to coordinate them. Then the samples are washed with solvent to eliminate the uncoordinated precursor materials [58, 59]. Besides, the improvement of effective strategies to combine high-quality functional MOF coatings with alloy substrate is highly desired to achieve multifunctional applications with sequential multiple-drug delivery and implant integration [60]. Surface protective coatings can be found in several applications such as medicine and aerospace. Organic coatings are often designed to protect a metal substrate from corrosion [61]. Nowadays, multifunctional organic coatings are in demand to improve surface performance. Due to the insufficient properties of coatings, the development of layers with nanoporous materials is known as a hot research area. Amongst, the porous nanoparticles with functional groups and organic–inorganic hybrid compounds with polymer matrices have attracted high attention [62, 63]. MOFs have a variety of flexible substituents to organic ligands, and the powerful coordination ability of metal ions is a considerable potential that guarantees for surface treatments to improve biocompatibility of implants. One of the significant challenges in the widespread utilization of MOFs is controlling their growth or deposition as thin layers on substrates [64]. Fabrication of a robust mechanical barrier via surface coating is an easy and efficient approach for coating substrates. Hence, it is a big challenge to achieve a surface coating with a robust and reliable mechanical and chemical barrier [65–67]. Generally, the medical layers should be safe, biocompatible, and biodegradable with nontoxic effects on the living cells [68, 69]. Coating metal implant surfaces with MOF coatings combines the chemical advantages of metal alloys with the compatibility of MOFs and the release of ions in the implant system. The development of fabrication and modified approaches can further decrease the toxicity of MOF coating and improve the chemical and mechanical stability. Researchers studied the various protective coatings available for the protection of alloys [70, 71]. For battery applications, MOF nanocomposites, including cobalt and cerium presented considerable effect as either the sulfur host or the coating interlayer for Li–S batteries [72]. In general, the most crucial advantage of MOF coatings is their poor chemical stability. The development of fabrication can further reduce the toxicity of MOF coatings and improve the stability properties. Application of the various coating strategies can achieve surface coverage by MOF coating materials to ensure medical performance and can be extended to different types of substrate surfaces. Some of the positive effects of biological coatings, such as anti-microbial activity against pathogenic bacteria, antioxidants and anti-inflammatory are important and can be included in orthopedic implants for natural modification of the surroundings. Several limitations related to the surface treatment of MOFs such as stability, homogeneity, maintaining porosity, the introduction of impurities, scalability, and compatibility with intended applications, are considered.

## Characteristics of MOF-coated surfaces

MOFs are porous materials that, when used as coatings on surfaces for biomedical aims, thin-film layer of MOFs offers a unique set of properties that enhance their performance. Biocompatible MOF-coated surfaces are an emerging research area with potential applications in various fields, including biomedicine, drug delivery, and tissue engineering. Biocompatibility is the most fundamental characteristic of the MOF-coated surface's ability to interact with living tissues without causing adverse reactions such as inflammation, cytotoxicity, or immune response [73]. Among other important characteristics, the hydrophobicity and hydrophilicity of the surface play an essential task in the performance of materials, particularly implanted devices. All living organisms and cells, bacteria can take place at the interface between an implant and the human body. Thus, an implant material was directed by researchers toward modifying implant material surfaces to obtain favorable host-material interactions [74]. The charge and hydrophobicity are two crucial surface properties that affect the bacterium-surface interaction [75]. The surface energy of a substrate is generally higher than coating materials to attain adhesion. The surface energy substrates can improve to increase uniformity, adherence, and durability. The concepts of hydrophobicity and hydrophilicity depend on the level of the surface energy of the substrate [74]. In general, the hydrophilic surfaces are osteogenic and they can enhance the activation time of several signaling pathways that directly or indirectly contribute to bone formation [76]. The origin of many infections is related to complex interactions between the pathogen and the implant surface. Therefore, the designing and manufacturing an infection-resistant coating is very challenging, and this coating must provide optimal broad-spectrum anti-microbial activity, protection against biofilm formation, and biocompatibility [77–79]. The contact angle measurements indicate the wettability (hydrophobic or hydrophilic) of a surface. Besides, the surfaces with a contact angle above 65° will be studied hydrophobic and below 65° hydrophilic [80]. At hydrophobic surfaces, the driving force is generally hydrophobic interactions that occur between the surface and the outer hydrophobic shell of most proteins. In addition, the driving force is generally the electrostatic interactions between the surface and the proteins [81]. The biomacromolecules can lead to the growth of MOFs in aqueous solution to give a strong coating that offers protection from environments that are usually destructive. ZIF-8 chemistry has suitability for bio-mineralization and its hydrophobicity [82]. The hydrophilic surface is also helpful for bone cell adhesion and proliferation to improve bone growth. For instance, the Mg-MOF-74/MgF_2_ coating is prepared on the AZ31B Mg alloy surface, and this coating has progressed the anti-corrosion and hydrophilicity of Mg alloys [83].

### Anti-microbial properties of MOF coatings

Infections caused by pathogenic bacteria are a severe threat to human health that results in seriously affects for human health [84]. Among traditional materials, silver, copper, zinc and gold-based inorganic metal nanoparticles have been proven to be effective anti-bacterial agents with broad-spectrum anti-microbial activity [85, 86]. Coating methods can offer new opportunities for providing topical therapeutic agents. Besides, the coating materials do not form a strong bond with the bone. Numerous antibiotics or anti-microbial peptides have been loaded onto implant surfaces for anti-bacterial treatments [87, 88]. Bacterial acid-responsive MOF coating on the implant surface can be fabricated for releasing large amounts of metal ions to kill pathogenic bacteria in the early postoperative period without affecting ossification in the later period [39]. For example, Mg^2+^ ions can induce an alkaline media to destroy pathogenic bacteria and are attributed to the regulation of inflammation [89]. Due to the emergence of new resistance mechanisms, anti-microbial resistance may disrupt the ability of the present medical care system. Therefore, the expansion of new bactericidal agents is necessary [90]. In general, bioactive MOFs act by releasing biologically active metal ions or ligands into the environment after the separation of metal–ligand bonds. MOFs should be made more stable using strong cross-links and post-surface modification to avoid toxicity to host tissue and microbes [91, 92]. Moreover, the anti-microbial activity of MOFs is related to the kind of metal in MOF structure, which can release metal ions gently as an excellent benefit compared to the metal/metal oxide NPs [93]. In general, anti-microbial agents can be bonded to the material surface via quaternary ammonium, imidazolium, or other groups [94, 95]. The Cu-SURMOF is used as marine anti-fouling coatings via the release of Cu^2+^ against marina bacteria for an anti-fouling protective layer, which is responsible for unwanted bacterial colonization of underwater surfaces [96]. The iodine-modified ZIF-8 coated surface demonstrates anti-bacterial activity against Gram-negative Escherichia coli and Gram-positive Staphylococcus epidermidis and Staphylococcus aureus [97]. The ant-biofilm performances of MIL-88B(Fe) and its hydrophobic variant MIL-88B(Fe)PA were compared, MIL-88B (Fe) loaded with IMI binds well to polystyrene surfaces and hinders Salmonella biofilm [98]. In another study, the Naringin-loaded MOF NPs for coating mineralized collagen were fabricated with the MOF structure. The release kinetics of Naringin could be controlled to improve osseointegration and evade bacterial infection [99]. Thin polymer using a MOF templating method, which contained porphyrin molecules, was synthesized and demonstrated PDT effects against E. coli [100]. The synergistic anti-bacterial activity was investigated by using the shrill edges of the GO sheets and the action of Co^2+^ ions released from GO/Co-MOF [101]. In additional work, NO was produced via the immobilization of MOFs on polymeric substrates with the deposition of Cu(II) CuBTC crystals onto the surface of carboxyl-functionalized cotton [102]. MOF thin films have shown promising anti-bacterial properties and have the potential to reduce bacterial infection due to metal ions or clusters coordinated organic ligands. Some MOF thin films can release anti-microbial agents such as silver ions, copper ions, or antibiotics, effectively killing bacteria and reducing infection. In addition, some MOF thin films can encapsulate and release antibiotics (or therapeutic agents( in a controlled manner, providing sustained and localized treatment for bacterial infections.

### Anti-corrosion properties of MOF coatings

**Fig. 3:**
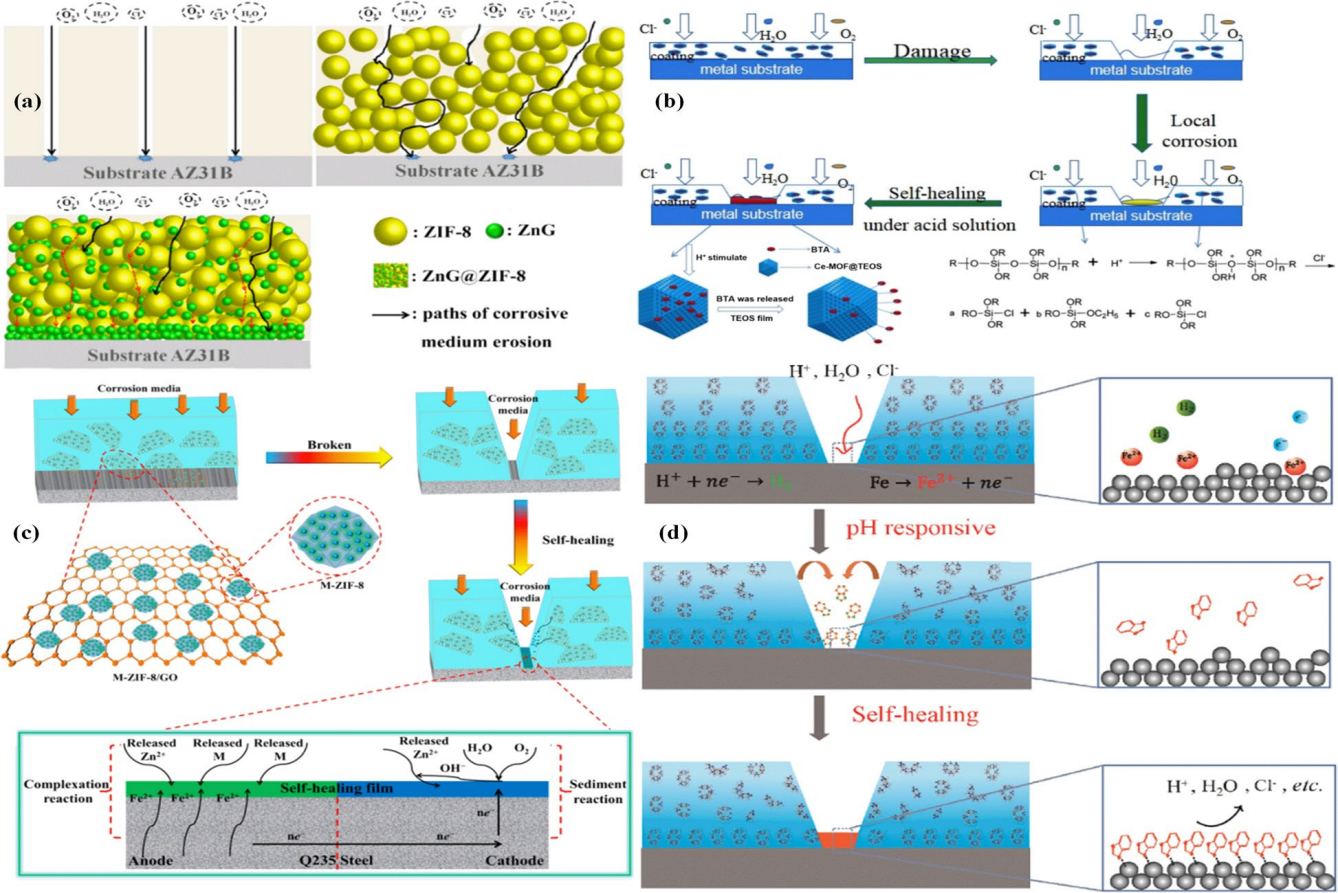
Schematic illustration of the probable anti-corrosion mechanism including EP coatings, ZIF-8/EP coatings, ZnG@ZIF-8/EP coatings (**a**) [112], the schematic of inhibitor loaded in nanocontainer as a self-healing coating (**b**) [113], schematic diagram of the anti-corrosion and self-healing mechanism in M- ZIF-8/GO/EP coating, diagram of coating damage and self-healing an enlarged diagram of the self-healing mechanism (**c**) [115], the pH-responsive release of the inhibitor from the epoxy coating containing ZIF-7 (**d**) [118]

The usage of protective anti-corrosion coatings is one of the most applicable ways to progress the corrosion resistance of metals. Metal corrosion significantly limits the use and development of medical metal-based materials due to its economic and medical disadvantages [103–105]. Applying MOFs in polymer coatings as a nano-container for packing corrosion inhibitors. MOF structure based on Ce ions and 1,3,5-BTC on graphene oxide (GO) nanosheets was fabricated and applied to make an epoxy-based anti-corrosion coating [106]. In other work, MIL-53 (Al) nanoparticles were prepared. Then the nanoparticles were incorporated in hybrid (tetraethyl orthosilicate + γ-glycidyloxypropyl trimethoxysilane) sol–gel coating as nano-filler, and the fabricated nanocomposite was used on Al 2024 alloy for superior barrier and protection properties [107]. The thermal stability of the sol–gel layer improved with the integration of MIL-53 (Al) MOFs. Silica sol–gel matrix incorporates Cu-1,3,5-tricarboxylate (Cu-BTC MOF) treated with sulfur compounds electrodeposited on Ni–Fe binary alloy substrate and used as a shielding coating. Cu-BTC MOF loaded with 2-aminobenzothiazol was fabricated by the encapsulation technique and inhibitor release has also been investigated by cyclic voltammetry [108]. In addition, one of the green, easy, and cheap methods for surface adjustment uses PDA for a comprehensive class of inorganic and organic materials [109]. ZIF contains zinc as a metal ion and imidazole or its derivatives, some of which have apparent corrosion inhibition against mild corrosion of acidic steel as an organic linker. The zeolite-imidazole (ZIF-67) of MOFs was fabricated on the GO platform that constructs ZIF-67/GO NPs for supplying polymer-based anti-corrosion coatings with both self-healing and barrier efficiency [110]. The ZIF-67/GO@APS composite could protect the surface of steel via mixed anodic/cathodic type (O_2_ reduction/Fe oxidation) mechanisms, and the corrosion of the iron sample was reduced. ZIF-8 has been the most widely investigated due to its easy fabrication, controllable size, excellent stability, and appropriate pore size. ZIF-8 nanoparticles were synthesized, and subsequently, superhydrophobic composite coating was fabricated by combining the super-hydrophobicity of ZIF-8/EP coating and the excellent bonding property of epoxy resin coating [111]. In general, MOFs have an excellent matrix for encapsulating corrosion inhibitors. MOFs-based anti-corrosive materials were loaded with zinc gluconate (ZnG) corrosion inhibitors (Fig. 3a), and next the ZnG@ZIF-8 composite anti-corrosive material are dispersed in an epoxy resin to fabricate film coatings and these coatings have excellent corrosion resistance compared with ZIF-8/EP coatings, EP coatings and ZnG + ZIF-8/EP coatings [112]. The Ce-based MOF loaded with benzotriazole with the controlled-release ability was fabricated for the simultaneous enhancement of the hydrophobic property and corrosion resistance of epoxy coatings [113] (Fig. 3b). ZIF-8 with rhombic dodecahedral structure interrelate with the matrix of various polymers and progress their thermal/mechanical properties due to organic ligands like imidazole [114]. Epoxy coatings containing enclosed MOF corrosion inhibitors, corrosive environment penetrates quickly from cracks and attacks MOF encapsulated in corrosion inhibitors. 2-Mercaptobenzimidazole was built in ZIF-8 on GO nanosheets and then embedded into epoxy coating to synthesize M-ZIF-8/GO/EP composite coating with pH-responsive and self-healing performances [115]. In another study, the dopamine was grafted on the surface of the MOF-5 and was combined into waterborne epoxy coatings [116]. The results indicate that the corrosion protection performance of epoxy coating with dopamine-MOFs (0.5 wt%) was higher than others. The mesoporous silica nanoparticle-benzotriazole- ZIF-8 (HMSN-BTA@ZIF-8) as corrosion inhibitor-encapsulated nanocontainer was fabricated using ZIF-8 as self-sacrificial template [117]. The synthesized material shows prominent pH-triggered activities in acidic and alkaline situations. The fabricated HMSN-BTA@ZIF-8 can covalently interact with epoxy, resulting in outstanding anti-corrosion and impressive self-healing properties. The loaded capacity of 2-Mercaptobenzimidazole on M-ZIF-8/GO was 9.12%, and the release trend of 2-Mercaptobenzimidazole and Zn^2+^ cations was synchronous (Fig. 3c). In ZIF-7 MOF, the linking benzimidazole is spatially separated by a framework as an inhibitor. ZIF-7 MOF with acid-sensitive coordination bonds can deliver the loaded active corrosion inhibitors to the desired location and enable the delivery of a more considerable amount of inhibitor. The intelligent dynamic polymer coatings with ZIF-7 for self-healing corrosion protection in acid conditions were studied by electrochemical impedance spectroscopy [118] (Fig. 3d).

## MOF coating for medical applications

In general, MOF modification can have significantly influence on its properties and performance in vitro and in vivo. Modifications of MOFs can change the surface properties, pore size, and chemical functionality of MOFs, which can influence their interactions with biomolecules and cells. In vitro, modifications of MOFs can alter their interactions with proteins, enzymes, and cells and affect the biocompatibility and toxicity of the materials. The surface modifications can improve the stability and selectivity of MOFs, making them valuable for medical applications such as drug delivery and biosensing usage. In vivo, MOF modifications can affect the pharmacokinetics and biodistribution of MOFs, altering their therapeutic efficacy and toxicity. The surface modifications can also improve MOF degradability and decrease immune responses. The surface-mounted MOF coatings have indicated considerable potential for several biomaterial and medical applications due to their unique properties. However, further research is needed to optimize their performance and ensure their biocompatibility and safety. There are several areas, such as medical implants, tissue engineering and drug delivery where surface-mounted MOF thin film coatings can make a remarkable impact in the medical field.

## MOF coatings for bone repair applications

**Fig. 4:**
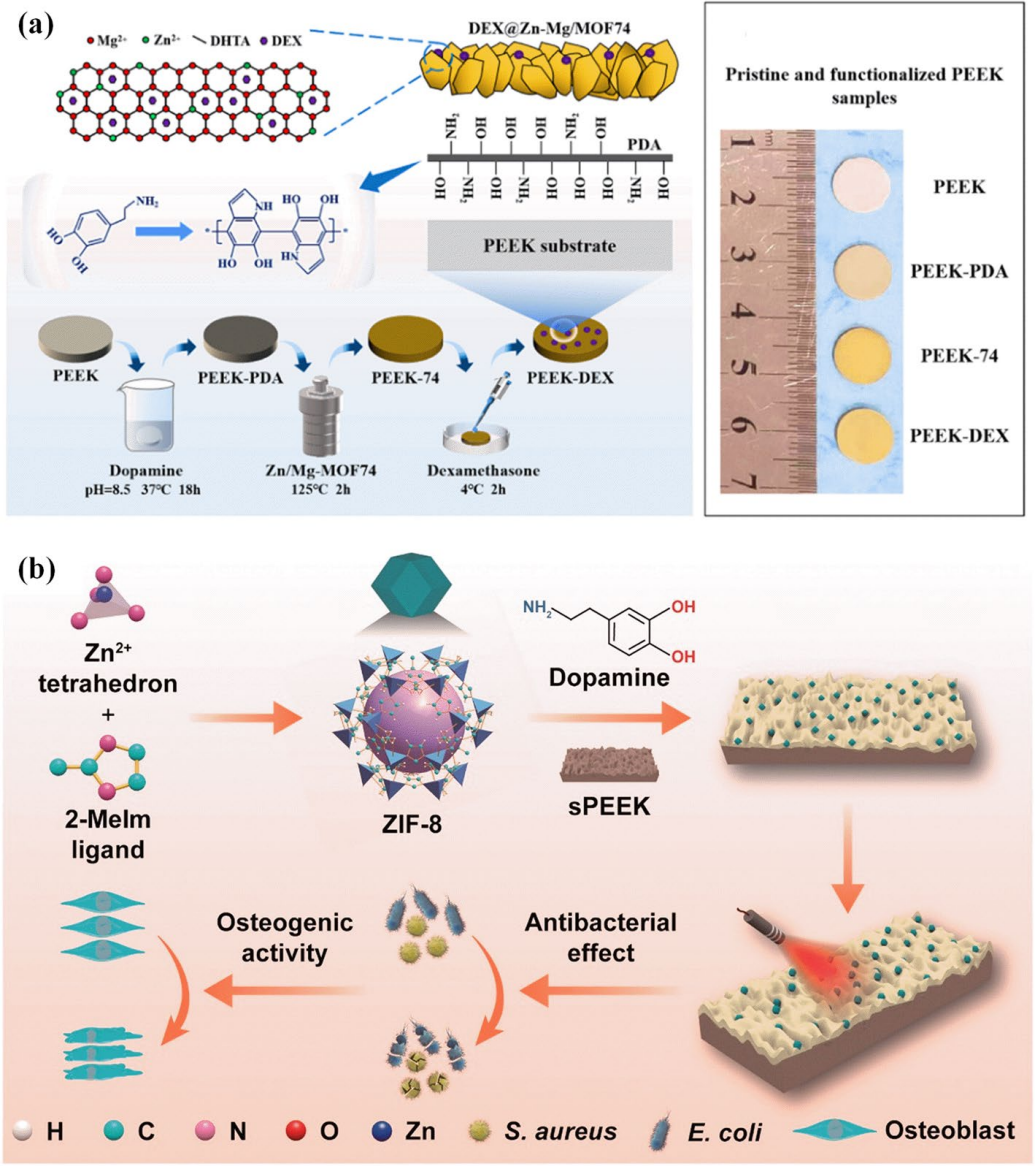
Preparation process of the DEX@Zn − Mg-MOF74/PDA composite and their physical pictures [15] (**a**) and the illustration reveals that the ZIF-8-inlaid pDA coating possesses photoinduced disinfection and osteogenicity for infectious bone repair [124] (**b**)

Nowadays, the need for implants to deal with bone problems is enhancing due to the increasing rate of population in the world. The implants are applied for various applications such as maxillofacial reconstruction, prolonged bone fracture fixation, hip replacement, knee replacement, correction of spinal fractures, osteoporosis, and orthopedics. The biofilms contaminate medical implants that spread infection is a severe public health concern [119]. The improvement of anti-bacterial influence on the implant by surface modification like loading with antibiotics and a combination of metal ions (Ag^+^, Zn^2+^, and Cu^2+^ ions) are performed [120, 121]. There is demand for developing better coating materials for implants with excellent biocompatibility and high functionality. The surface modification of the implants or coating of some MOF on the implant’s surface can eliminate the infection and also kill the microorganisms. In general, the change of implant surfaces with MOF can have a significant influence on their performance in vitro and in vivo. Laboratory studies have revealed that MOF coatings on implant surfaces can enhance cell adhesion, proliferation, and differentiation, improving bone integration and tissue regeneration. Besides, the porous structures of MOFs allow for the incorporation and controlled release of drugs, growth factors, and other therapeutic agents, further increasing the bioactivity of the implant. Polyetheretherketone (PEEK) as elastic modulus is a highly biocompatible, with a low coefficient of friction and it also can be used in bone [122]. The clinical applications of PEEK remain due to insufficient osteogenic activity and anti-infective capacity [123]. The dual-metal − organic framework (Zn − Mg-MOF74) coating is bonded to PEEK, and then dexamethasone is loaded on the coating surface. The release of Mg^2+^, Zn^2+^, and 2, 5-dihydroxyterephthalic acid, and the formation of an alkaline microenvironment on the coating surface leads to high anti-bacterial properties against E. coli and S. aureus (Fig. 4a). The dexamethasone-Zn − Mg-MOF74 coating-modified PEEK implant demonstrated bacteriostasis, angiogenesis, and osteogenesis properties [15]. In another study, the polydopamine-wrapped ZIF-8 coating on the sulfonated polyetheretherketone (PEEK) was fabricated [124]. The coating shows controllable Zn^2+^ releases and great photothermal capacity under near-infrared (NIR) irradiation. In addition, the layer possesses bactericidal efficiencies of 100% against Staphylococcus aureus and Escherichia coli under NIR irradiation in vitro (Fig. 4b). In general, in vivo studies have revealed that MOF-coated implants enhance bone formation and implant stability compared to uncoated implants. The presence of MOF can also decrease inflammation and minimize the risk of infection by promoting the growth of beneficial bacteria and preventing the colonization of harmful bacteria.

### Magnesium alloys

**Fig. 5:**
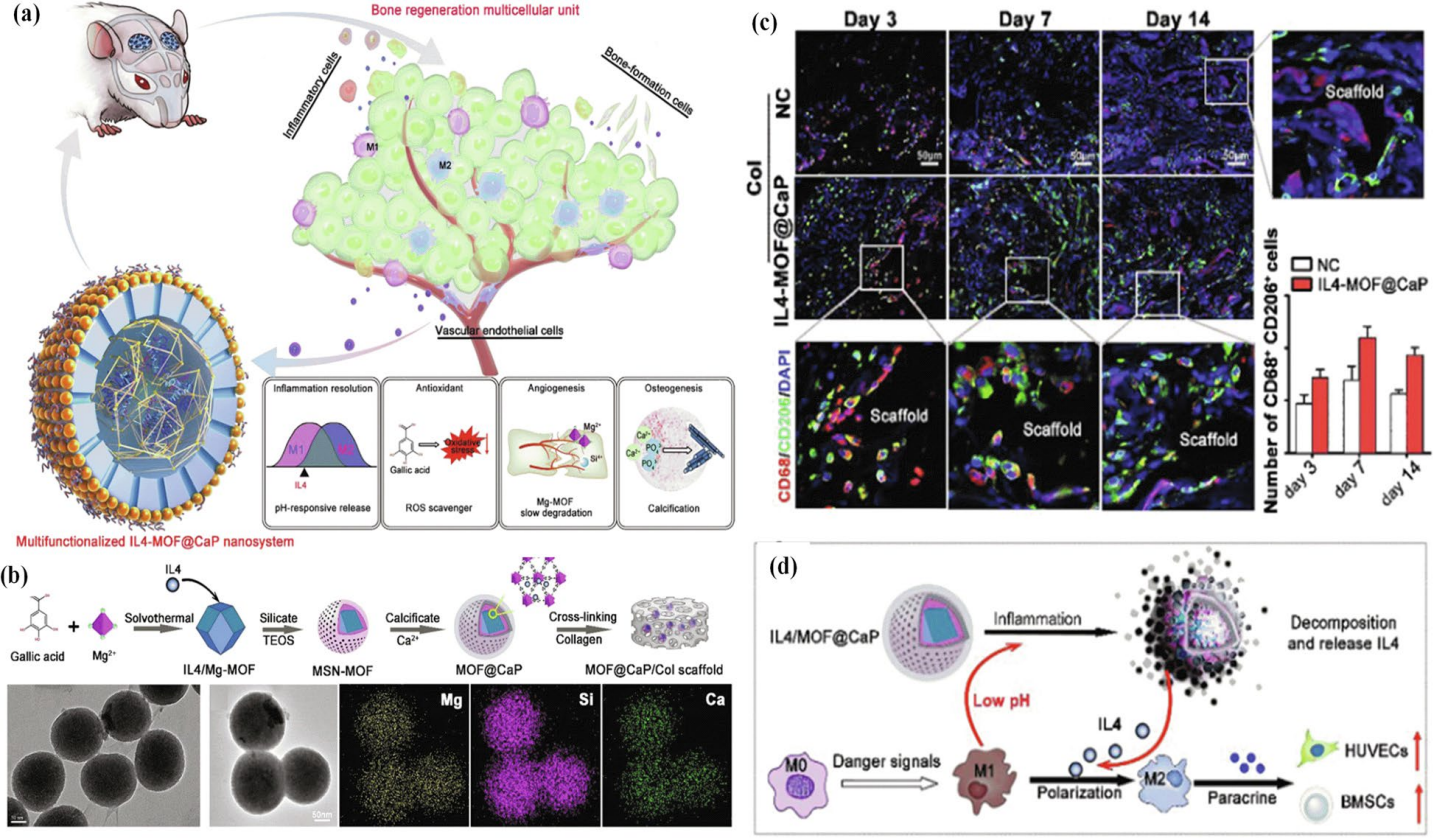
Schematic illustrations of IL4-MOF@CaP nanosystem for enhanced bone regeneration (**a**), Schematic illustration of the synthesis and characterization of IL4-MOF@CaP (**b**), In vivo spatiotemporal evaluation of the M2 macrophage polarization around the Col or IL4-MOF@CaP/Col scaffolds [blue, green and red respectively present DAPI, CD206, and CD68 Staining] (**c**), Schematic illustration of the accurate and proactive immunomodulation properties of IL-4-MOF@CaP, which was beneficial for angiogenesis and osteogenesis (**d**) [129]

In general, the biodegradable magnesium (Mg) and its alloys have been extensively considered as major candidates for orthopedic implants due to their unique properties such as sufficient mechanical strength and Young's modulus comparable to human bone [125, 126]. However, Mg alloys tend to lose ions and are highly susceptible to corrosion [127]. It is noted that Mg ion stimulates new bone formation but the rapid degradation and adverse outcomes associated with these implants are insurmountable barriers to clinical application [128]. The biocompatible gallic-acid-magnesium-MOF@calcium phosphate nanoplatform is synthesized by Wang’s group and is used for osteogenic nucleation site formation by providing a preferable repair microenvironment for bone regeneration multicellular units. IL4-MOF@CaP demonstrated an excellent proactive and accurate immunomodulatory influence [129]. It also includes magnesium for angiogenesis, gallic acid to eliminate reactive oxygen species, calcium and phosphate to ensure bone matrix calcification. The results indicated that the cranial bone defects in the IL4-MOF@CaP/Col group were regenerated into the best form (Fig. 5a-d). In general, modifying implant surfaces with MOF has an excellent potential to progress the efficiency of medical implants, especially in orthopedic and dental applications.

The advantage of using MOF thin films is their ability to store and release bioactive molecules such as growth factors and antibiotics. The porous structure of MOFs allows the controlled and sustained release of these molecules, which can increase cell growth and tissue regeneration. The biosafety of micron/nanoscale Mg-MOF74 followed by an assessment of early osteogenesis and angiogenesis promotion, is studied [130]. The nanoscale Mg-MOF74 demonstrated lower in vitro cytotoxicity and in vivo cardiotoxicity compared to the micron-scale treatment groups and also showed outstanding results in the angiogenesis simulation experiment. The multi-functional Mg-phytic acid (PA)&zoledronic acid (ZA) metal organic complex coating on biodegradable Mg was synthesized, and subsequently, used to perform the corrosion controlling and differential regulation on osteoblast vs. osteoclast responses simultaneously [131]. In addition to electrochemical corrosion resistance and long-term degradation behavior, this coating demonstrated considerable promotion and suppression of the growth of pre-osteoblasts and osteoclasts, respectively. Various polymeric materials can be used for coating Mg alloy for bio-implants, including poly(lactide-co-glycolic) acid, poly (glycolic acid), polycaprolactone, polylactic acid, chitosan, collagen, fibrin and alginate [46]. Among these polymer coatings, polycaprolactone (PCL) stands out due to its unique properties [132]. In other work, HKUST-1 MOF was modified with folic acid and incorporated into the polycaprolactone matrix, which was further fabricated on the surface of AZ31 Mg alloy [133]. The Mg-PCL-MOF coating showed excellent corrosion resistance and enhanced the viability and differentiation of osteogenesis cells by the release of Cu^2+^ ions from MOF-FA during the degradation process.

### Titanium-based alloys

**Fig. 6:**
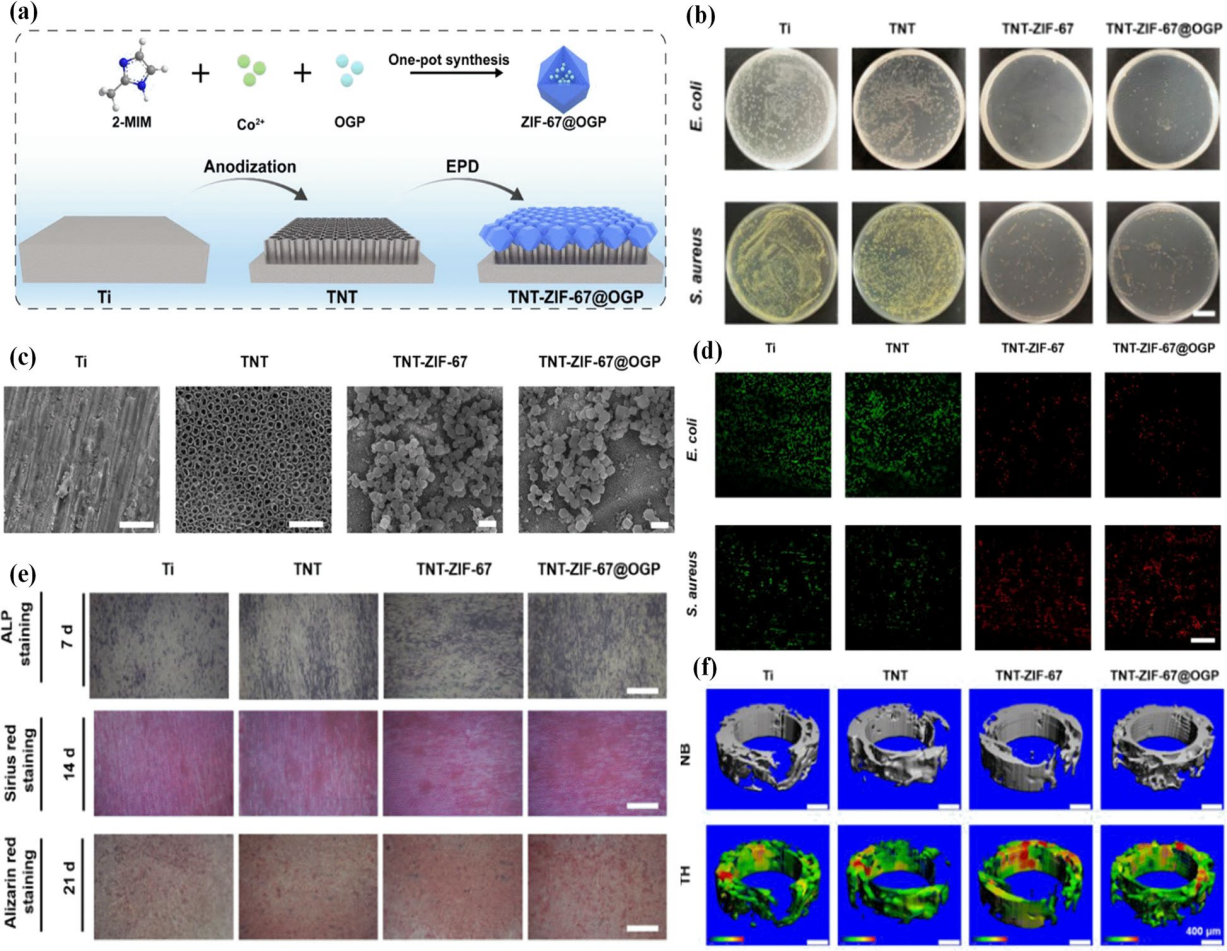
Schematic illustration of the fabrication of the TNT-ZIF-67@OGP sample (**a**), the evaluation of anti-bacterial activities of the TNT-ZIF-67@OGP sample in vitro (**b**), SEM images of Ti, TNT, TNT-ZIF-67, and TNT-ZIF-67@OGP (**c**) Live/dead fluorescence images of E. coli and S. aureus after incubation with multiple samples (**d**), Representative images of ALP, Sirius red, and Alizarin red staining of MSCs cultured onto various samples, scale bar: 200 μ m (**e**), New bone formation quantified by micro-CT after surgical implantation for four weeks (**f**) [149]

In general, titanium (Ti) and its alloys indicate significant potential for complex tissue repair and bone substitutes due to their feasible mechanical features, suitable corrosion resistance, and good biocompatibility [134]. MIL is a kind of MOF used in various research due to its high surface area and stable structure that can bond Ti^4+^ as the coordination center and 2-aminoterephthalic acid as the ligands [135, 136]. Ti-MOF nanofibers were prepared using an electrospinning method and they showed a new strategy for fabricating dental nano-coatings with high surface area and thermal stability properties [137]. ZIF-8@levofloxacin nanoparticle deposition and (gelatin/chitosan)_5_ multilayer coating on Ti substrates were synthesized and used to release levofloxacin (Levo) and Zn^2+^ ions [138]. The fabrication of MOF@Levo/LBL improved in vitro adhesion, proliferation, and differentiation of osteoblasts and showed strong anti-bacterial ability against Escherichia coli and Staphylococcus aureus. In another study, the template-directed preparation of Co-based ZIF-67-derived was fabricated by annealing treatment of ZIF-67 grown on the nickel/titanium alloy fiber substrate [139]. The ZIF-67-derived coatings indicated the tremendous mechanical stability, superior solvent resistance, and highly efficient extraction performance for polycyclic aromatic hydrocarbons compared to the commercial polydimethylsiloxane fiber. The earth elements such as Nd have been applied to progress the strength, flexibility, and corrosion resistance of alloys [140, 141]. The amorphous NH_2_-MIL-125-Ti contains spherical metal–organic framework analogue nanoparticles with neodymium ions doped [142]. This material shows the corrosion resistance and anti-bacterial properties of the bare Ti base. In other work, the anionic amphiphilic gold nanoclusters -based mixed-metal metal − organic network films on titanium disks were synthesized by Zhang groups, and they showed anti-bacterial activity due to the protonation level of pMBA ligands and increased hydrophobic interactions synergistically reinforcing the disorders of the bacteria [143]. Osteogenic drugs such as dexamethasone are used in implants to support the bone differentiation of human mesenchymal stem cells. They are also relatively longer half-life, more cost-effective, and more affordable than growth factors [144, 145]. The dexamethasone@zeolitic imidazolate framework-8 (DEX@ZIF-8) nanoparticles were immobilized into the micrometer-scale artificial etch pits on the Titanium (Ti) disc by using the methanol-induced regenerated Silk fibroin membrane encapsulation which has been studied by Tong’s groups [146]. Due to the barrier influence of the ZIF-8 shell and the Silk fibroin membrane, this implant could release dexamethasone in a controlled manner and also had good cytocompatibility with MC3T3-E1 cells in vitro cell culture. Ti-based MOFs doped by Nd^3+^ ions exhibit suitable biocompatibility in the human body [147]. Besides, hydroxyapatite, as the main inorganic constituent of human bones and teeth, has great osteoconductivity and osteoinductivity with high biocompatibility. Zhang et al. [148] prepared a regular array of TiO_2_ nanotubes using anodic oxidation on the surface of titanium-based, and subsequently, deposition of ZIF-8@Nd and hydroxyapatite composite coatings by using an electrochemical deposition approach was performed. They investigated the biotoxicity, anti-bacterial activity, and corrosion resistance of the present layer to do more research on the implantation of Nd^3+^ ions implantation in vivo. In another study, the pH-sensitive ZIF-67@ osteogenic growth peptide nanoparticles modified titanium dioxide nanotube substrates was fabricated to inhibit bacterial adhesion and improve bone-implant osteointegration [149]. These implants showed strong anti-bacterial activity against E. coli, S. aureus, S. mutans, and MRSA. Besides, they demonstrated potential anti-bacterial pathways like ROS, and reduction of ATP level. These properties are due to the hydrolysis of ZIF-67, the release of cobalt ions and also the formation of an alkaline microenvironment (Fig. 6a-d).

### MOF coatings for drug delivery applications

MOFs have received high attention for encapsulating functional guest species for increasing drug delivery. Drug release rates are closely related to host–guest interactions between drug molecules and the MOF framework [83, 150, 151] They can control the guest species that move between their interior and exterior structures by modifying mesoporous MOF structures. Due to the special surface area and high porosity and particularly the biological application of central ions, MOFs have become one of the most developed drug delivery systems [152]. Therefore, the release kinetics of different drugs can be controlled with the help of MOF nanocrystals loaded in the coatings [99]. The release for osteogenic drugs had great potential for regenerating vital bone defects. A small number of growth factors or osteogenic drugs can facilitate ossification, but uncontrolled release of these reagents into the body can cause undesirable side effects [144]. Ran et al. [146] fabricated dexamethasone delivery platform on Ti disc by immobilizing dexamethasone@ZIF-8 nanoparticles into the micrometer-scale artificial etch pits on Ti substrate by using methanol-induced regenerated silk fibroin membrane encapsulation. ZIF-8 coatings on Ti substrate can further serve as a platform for controlled drug delivery was demonstrated. Besides, in vitro cell culture assay showed that the silk fibroin -DEX@ZIF-8-Ti had good cytocompatibility with MC3T3-E1 cells. Drug eutectic is a single-phase crystalline compound that improves the physical and chemical properties of drugs by altering the solid properties of active drug molecules [153]. In the study, MOFs with curcumin as a ligand and Zn^2+^ as the center were fabricated by a eutectic method. The controllable double-release system of curcumin and Zn^2+^ for diabetic wound repair was studied by loading the MOFs into the hierarchical micro/nanofibrous poly (L-lactic acid) (PLLA) scaffolds [154]. Modifying the MOF surface in vitro conditions can enhance drug stability, solubility, and bioavailability and improve its targeted delivery to specific tissues. It can also reduce drug toxicity by controlling the release rate and preventing degradation. Besides, the modified MOFs can improve the efficacy of combination therapies by delivering multiple drugs simultaneously by releasing the drugs in a sequential manner to achieve a synergistic influence. Modification of the MOF surface in vivo conditions can improve the pharmacokinetics and biodistribution of the drug and lead to better therapeutic results. They can also decrease drug side effects by targeting particular tissues and minimizing exposure to healthy cells. In addition, the modified MOFs can improve a drug's ability to cross biological barriers such, as the blood–brain barrier, enabling it to reach formerly inaccessible goal sites.

**Fig. 7:**
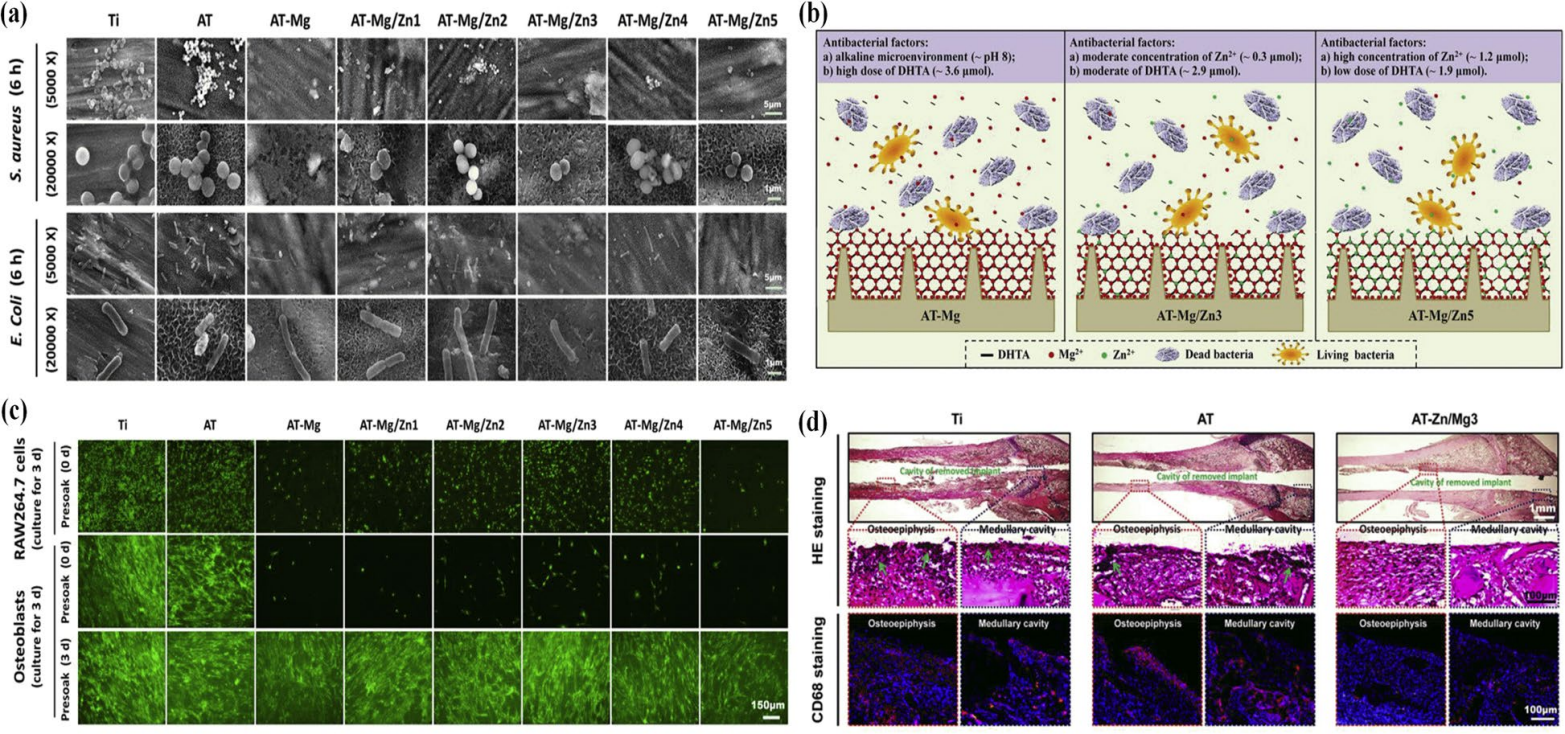
SEM images of bacterial morphology for both S. aureus and E. coli after on various substrates (**a**), illustration scheme of potential anti-bacterial mechanisms of different substrates (**b**), FDA fluorescent images of living RAW264.7 cells and osteoblasts after culturing for 3d on various normal or presoak (samples were soaked for 3d in PBS solution before cells incubation) substrates (**c**), HE immunohistochemistry (red: endochylema; blue: nucleus) and CD68 fluorescence (red: CD68 protein; blue: nucleus) staining images of different implantations (**d**) [39]

The use of a protective coating can provide new tools for the storage, handling and transport of drugs in drug therapies [155]. Clinical statistics show that infections associated with prosthetics and implants are generally the most common cause of complications. The strong bactericidal capacity of implants in the early stages of implantation increases the success rate of revision surgery due to bacterial infection [39]. Thus, the new ways of generating thin film coatings for sequential delivery of antibiotics and osteoinductive growth factors from surfaces applying the conformal nano-scale coatings [156, 157]. The porosity, stable structures, great degradability and hypotoxicity of MOFs make them best possible for drug carriers. These nanoparticles, as novel agents, are used for therapeutic drug delivery and multifunctional surface modification implants that change with the effector cell type [158, 159]. Orthopedic implant research usually focuses on promoting bone integrity and preventing infection. The multifunctional mineralized collagen coating on titanium implant with the aid of MOF nanocrystals was synthesized to control the release of naringin as herbal medicine could improve osseointegration and inhibit bacterial infection [99]. In another study, the magnesium/zinc-metal organic framework (Mg/Zn-MOF74) coating on an alkali-heat treated titanium surface was fabricated for anti-bacterial, anti-inflammation and pro-osteogenesis properties [39]. This hybrid showed good stability due to the content of Zn^2+^ ions. It demonstrated strong anti-bacterial ability due to the degradation of MOF74 coating and also displayed good early anti-inflammatory properties to native titanium substrates (Fig. 7a-d). In general, modification of MOFs for drug delivery can considerably enhance the therapeutic potential of drugs and make them more efficient and safer for patients.

### Other potential medical applications

Several potential applications for MOF coatings could provide promising medical results. Thrombosis is a significant cause of thromboembolic disorders that affect many people worldwide. Antithrombotic agents are one of the plants that used for the treatment of thromboembolic diseases. Traditionally, these drugs acting as vasodilators are administered to prevent artery occlusion. Even in some cases, the drug stents are implanted in an artery to prevent it from becoming permanently blocked [160]. Cu-based MOFs have been considered as a component in antithrombotic coatings for cardiovascular implants. The pore size of MOFs is significantly smaller than that of polymer structures, resulting in drug loading and stable release using preset nanoparticle coatings [161]. MOF / polymer composite materials for nitric oxide release are known as suitable clues for new planting materials [162]. Chronic non-healing wounds are a vital issue for people with diabetes. Therefore, it is desirable to create a multifunctional coating to save the wound from inflammation, but also to accelerate cell proliferation and tissue regeneration [163, 164]. Cu-MOFs are unstable in solutions containing physiological proteins and prevent their direct use in wound healing. The folic acid-modified HKUST-1 showed decreased cytotoxicity and increased cell migration in vitro [165, 166]. However nitric oxide (NO) as a gaseous drug has been used to heal diabetic wounds. The electrospun scaffold with NO@HKUST-1 incorporated as a controllable NO-releasing vehicle was studied [162]. The selective coating on the surface of sensors is in high demand to improve the sensitivity and selectivity of biosensors. In particularly, MOF coatings are very effective in preserving the biological detection capabilities of antibodies immobilized on sensor surfaces [167, 168]. The emergence of MOF (ZIF-8)-based biosensors has led to the idea of in situ biochips. The ZIF-8 coated biochips retained over 80% of recognition capability after one day (80 °C) [169]. The label-free LPG biosensor based on a thin film of ZIF-8, with encapsulated glucose oxidase (GO_x_), was studied. The ZIF-8/GO_x_ coating shows good reusability for detecting glucose [170]. The ZIF-8/GOx modified LPG fabricated by femtosecond fiber laser exhibits high sensitivity to surrounding refractive index. The wavelength shifts are correlated with glucose concentrations (1–8 mM) with a response coefficient (0.5 nm/mM). The fabricated multifunctional polydopamine-coated MOF biosensors were studied for detection of miRNA-122 with Zn^2+^ -triggered aggregation-induced enhancement and synergistic chem-photothermal treatment in vitro [171] and it has potential in the application of correct diagnosis and synergetic therapy of cancer. In another study, the sensitive fluorescent biosensor based on polydopamine-coated Zr-based MOFs (PDA/UiO-66) was studied for adenosine triphosphate detection [172]. In general, MOF surface modification can have significant effects on the performance and biocompatibility of surfaces, making them promising materials for various biomedical applications. However, more research is needed to optimize MOFs for particular medical applications, and to evaluate their biocompatibility and long-term safety.

## Techniques of surface-coated MOFs

To date, various techniques have been expanded depositing MOF thin films on substrates. These techniques can significantly improve the use of MOF feedstock and simplify film synthesis. Making layers or membranes by depositing MOF nanoparticles can be performed by popular methods like in situ growth, dip coating, spin coating, spray and electrospray coating, layer-by-layer and liquid phase epitaxy methods, vacuum and gas phase deposition, electrochemical deposition of MOFs. In general, most processes are effective in obtaining coatings but require high temperatures. Other approaches have long synthesis times or colloidal methods or require specialized equipment [173].

### In situ growth of MOFs

**Fig. 8:**
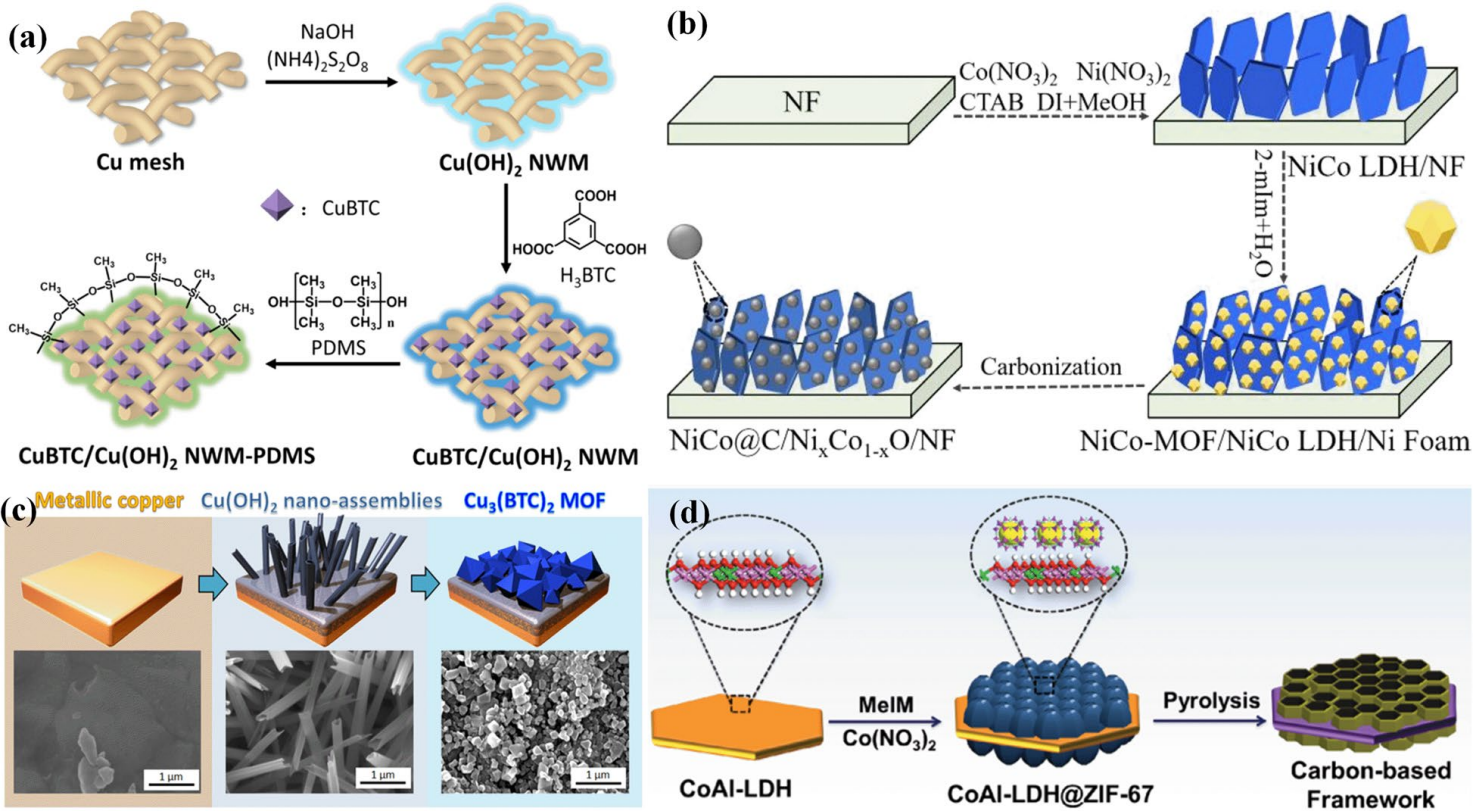
Schematic preparation process of superhydrophobic Cu-MOF based copper mesh (**a**) [184], the fabrication of the NiCo@C/Ni_x_Co_1-x_O/NF hierarchical composite from the NiCo-MOF/LDH/NF template (**b**) [185], Illustrations and respective SEM images during the conversion process from a metallic copper into Cu_3_(BTC)_2_ MOF thin film via a formation of Cu(OH)_2_ nanotube assemblies (**c**) [187], illustration for the synthesis of porous honeycomb-like carbon-based framework (**d**) [188]

The in-situ growth is the most common MOF coating method that requires continuous heating of a synthetic solution containing a metal compound and an organic ligand [174]. The in situ growth technique works by seeded growth of MOFs on the surface of the substrate for the formation of MOFs within the matrix, which sometimes shows better performance than other related methods [175]. In addition, suitable arrangement can be achieved by finding the particular conditions under which the direct growth of MOFs on substrates is obtained. In this method, MOFs are synthesized on the surface of the substrate by the dissolution of metal salts and organic ligands into such solvents as water, ethanol, DMF, DMSO, and benzene, followed by hydro/solvothermal reaction in a pressurized reactor containing the substrate immersed in that vessel [176, 177]. A microwave-assisted deposition is an alternative technique for in-situ rapid growth similar to the above-mentioned method, but using microwave energy as a heating source [178]. It is noted that the properties of MOFs are controlled by changing factors like temperature, concentration, and solubility of the reactants, including metal salts, ligands and pH of the solution [179, 180]. In general, the simplicity of the in-situ growth makes this method an interesting approach. Metal substrates like Cu(II) or Zn(II), may act as a source of metal ions for the synthesis of the MOF film by dipping into the corresponding ligand solution [181]. For instance, the in situ post-conversion growth of an HKUST-1 film on a metallic copper substrate was studied [182]. It seems that the in-situ growth method is clearly a more straightforward and faster procedure than other synthetic methods for the thin film growth of MOFs. Notably, the concentration of metal ions near the substrate surface can affect the nucleation and growth rates of MOF films. The growth process of Zr-MOFs on fiber was performed by MOF-808 as a model framework due to the high chemical stability [183]. Then UiO-66-NH_2_ is coated on PET fiber as another Zr-based MOF. These MOF-coated textiles exhibited the most increased activity for the hydrolysis of a nerve agent simulant DMNP and a nerve agent GD. A hydrophobic Cu-MOF-based copper mesh was fabricated by in-situ growth of Cu-MOF on a copper mesh substrate containing Cu(OH)_2_ nanoarrays followed by treatment with methylpolysiloxane (Fig. 8a) [184]. The as-synthesized MOF on modified Cu mesh showed great thermal and chemical stability as well as high efficiency for the separation of various oil–water mixtures. The NiCoLDH/NF precursors have been utilized for in situ fabrication of NiCo-MOF on NiCoLDH surface in the presence of imidazole-based ligands (Fig. 8b) [185]. NiCo alloy@C/Ni_x_Co_1−x_O/NF hierarchical composite obtained by after subsequent carbonization of this material, the obtained NiCo alloy@C/NixCo_1−xO_/NF hierarchical composite demonstrated excellent high electrocatalytic activity for oxygen evolution reaction. Fabrication of MOF nanofibrous membrane has been reported fabricated by in-situ growing H3PW12O40 (PW12) @UiO-66 crystals onto crosslinked polyacrylic acid-poly (vinyl alcohol) nanofibers fabricated via electrospinning technique [186]. The fully oriented copper-based MOF thin films were also prepared by the epitaxial growth approach, using oriented Cu(OH)_2_ nanobelts as a sacrificial template (Fig. 8c) [187]. In another report, the well-defined carboneous architecture is obtained by in situ nucleation and directed growth of MOFs arrays on the surface of LDHs nanoplatelets,and subsequent pyrolysis treatment, which resulted in the formation of electrocatalysts for the oxygen reduction reaction (Fig. 8d) [188].

### Dip coating of MOFs

Dip coating refers to immersing, or dipping, a substrate into a solution of containing coating material at a constant speed. After draining, the coated piece as a thin film can then be dried. This technique can be used for pre-formed MOFs by applying surfactant-assisted dip-coating to prepare a flexible three-dimensional porous composites [189, 190]. In the dip coating method, metallic and organic precursor solutions must be mixed well to form a uniform colloidal suspension. A substrate is placed in the colloidal suspension to obtain a MOF coating. This method is relatively fast for depositing MOF crystals through dip-coating that permits reasonable control over the film thickness in the range of 40 nm to 1 μm. The film thickness depends on the withdrawal speed of the dip-coater, the number of coating MOF layers and the washing and drying among each step [191, 192]. The benefit of this technique is that they can be fabricated comparatively quickly and their thickness can be nearly designed. They seem to show poly-dispersed crystallinity comparison with the layer-by-layer technique [193]. The chromium MIL-100 (Cr) MOF nanoparticles were prepared high optical quality thin films by dip coating deposition [194]. In other work, the crack-free MIL-101(Cr) and MIL-101(Cr)-NH_2_ MOF films on alumina were synthesized by a dip-coating method in the presence of polyethylenimine [195]. S. Nadar et al. [196] have embedded α-amylase ZIF-67 MOF by single pot onto the melamine sponge using surfactant assisted dip coating method. They showed that the encapsulation could keep the active conformational structure of α-amylase after the immobilization procedure with excellent bioactivity. MOFs ZIF-8 films on silicon substrates were synthesized by a dip-coating method and the thickness of the ZIF-8 film is controlled by the number of growth cycles [197]. Weng’s group applied a one-step approach to immobilize nano Cu-MOF crystals onto cardiovascular stents by using polydopamine as the linker and coating matrix [198]. The biocompatible coating on stents is used for simultaneous in situ catalytic generation of nitric oxide and delivery of copper ions in order to improve anti-coagulation and anti-hyperplasia abilities. LDHs as a class of layered inorganic materials, have provided a cost-effective and environmentally friendly way to prevent corrosion of metal substrates. The superhydrophobic ZIF-8/PVDF/LDH double-layered coating on the AZ31B Mg alloy was fabricated through electrodeposition and dip-coating approaches for usage in corrosion protection. In this coating, the LDH structure not only acted as a protection shield but also strengthened the binding force between the substrate and the top superhydrophobic ZIF-8 layer [199].

### Spin coating of MOFs

**Fig. 9:**
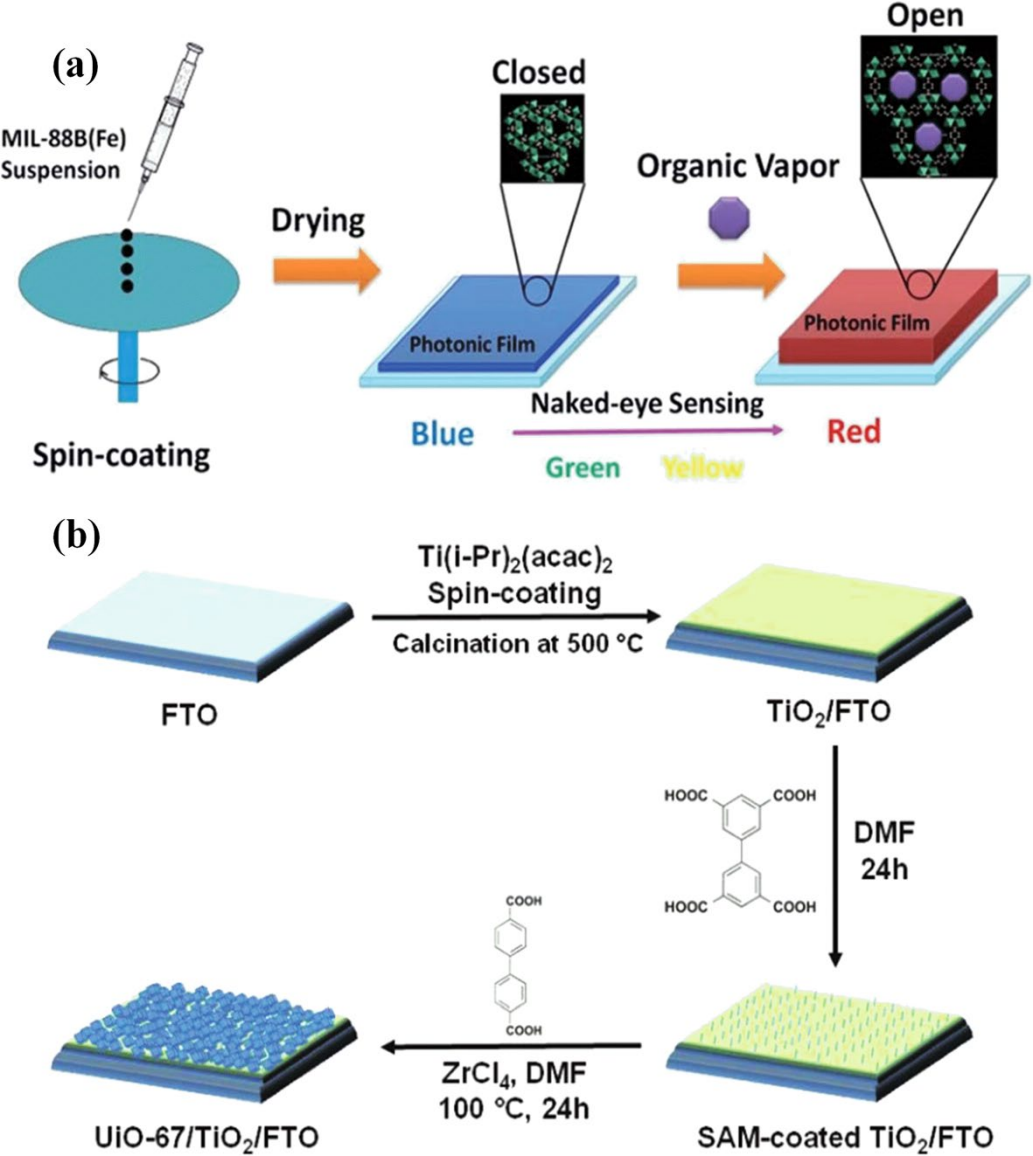
Schematic of the fabrication of NH_2_ -MIL-88B photonic films on silicon wafers, and exposure to organic vapors (**a**) [204], Schematic of the fabrication generic methodology of MOF/TiO_2_/FTO film (**b**) [205]

It is notable that the success of the spin coating method is controlled by several important factor’s rotation of the fluid distribution, stable fluid outflow, and finally drying by evaporation. The controlled evaporation and flow control stages have the greatest impact on the final coating thickness [200]. The ultrathin selective layers prepared from the spin-coating demonstrate great separation performance with reduced material cost [201]. One obvious disadvantage of spin coating deposition method is that the films containing MOFs are not rigidly bound to the substrate. This approach not only enables MOF thin films to be functionalized, but also removes the usage of strong chemical solvents, protecting the MOF thin films from corrosion or peeling off from the substrate [202]. For other work, the dual-layer of hydrophilic calcium alginate layer and a porous 2D covalent organic framework layer via the spin-coating assembly with a thickness of less than 100 nm was fabricated on a porous hydrolyzed polyacrylonitrile substrate and leads to superior water-selective performance with permeation flux [203]. Generally, photonic films show an appropriate light response to organic vapors. The NH_2_ -MIL-88B photonic films were fabricated by the simple spin-coating technique with the potential as application in ultraviolet–visible reflection analysis and an optical digital camera, revealing the selective response to different organic vapors (Fig. 9a) [204]. In other work, MOF thin films on TiO_2_-coated conductive glass substrates were fabricated and applied for an efficient catalyst for the photo-oxidation of thioanisole (Fig. 9b) [205]. The flexible NH_2_-MIL-88B MOF-based one-dimensional photonic crystal was synthesized by a spin-coating approach. It was used as the intrinsic functional layer due to its selective breathing behavior upon exposure to different guests [206]. For another application, the ultrathin mixed matrix membranes containing 2D CuBDC-ns MOF nanosheets as fillers are prepared by using the spin coating method and subsequently used for the separation performance of the CO_2_/CH_4_ mixture [207]. In addition, measuring and monitoring CO_2_ levels by using MOF coatings for areas like food packaging and indoor human safety is essential. This sensor is fabricated via self-assembly of a transparent film of ZIF-8 MOF nanoparticles by using the spin-coating technique.

### Layer-by-layer methods

**Fig. 10:**
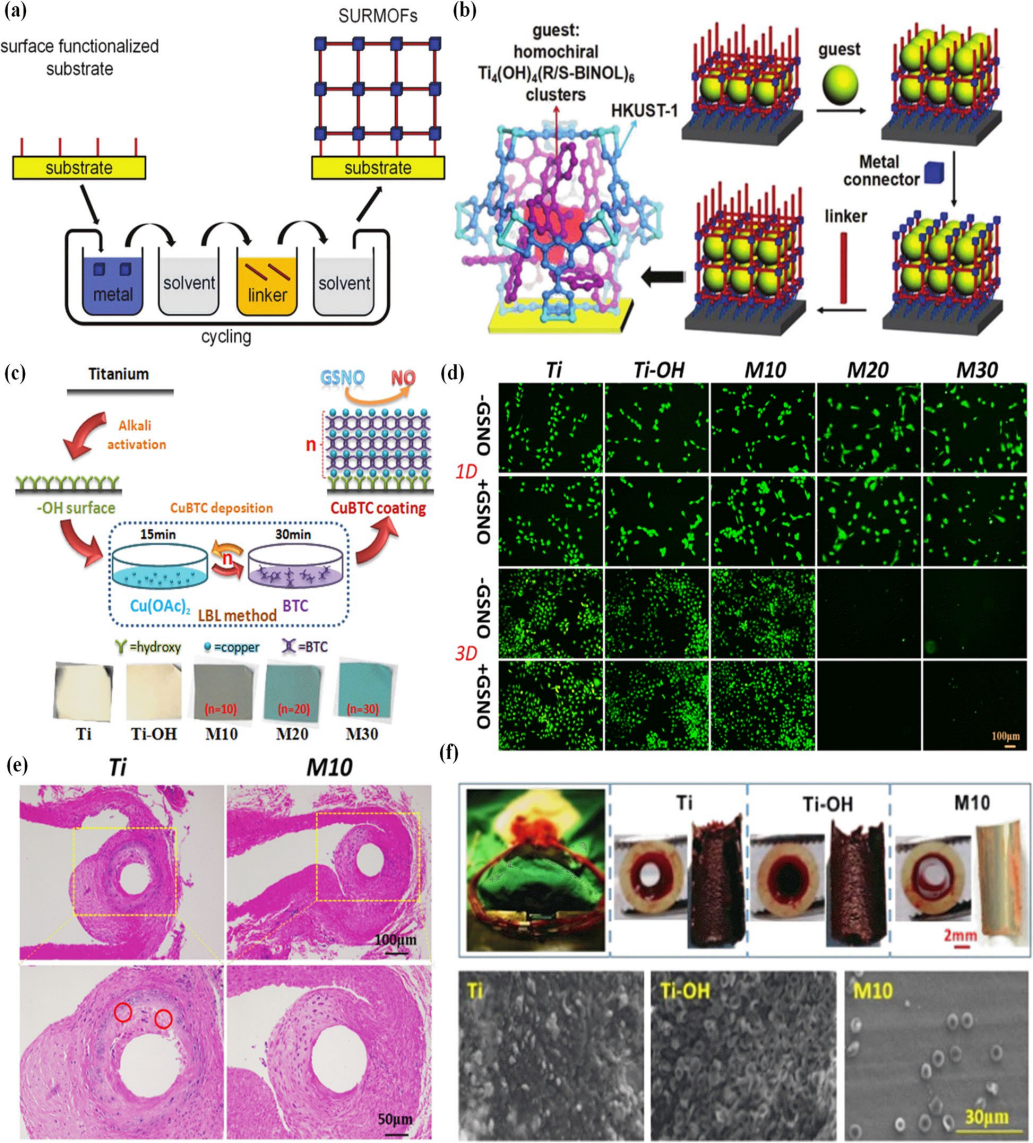
The LBL assembly technique for the growth of surface-mounted metal–organic frameworks (SURMOFs) (**a**), Schematic presentation of the flexible encapsulation of guest species (**b**) [210], schematic diagram of LBL deposition of CuBTC coating on alkali-activated titanium surface and optical micrographs showing the sample color change (**c**) The proliferation of EC with and without the addition of NO donor (**d**), Implantation of naked and CuBTC-coated titanium wire in abdominal aortas of SD rats for four weeks (**e**), Thrombogenicity of the samples in an arteriovenous shunt model of New Zealand rabbit and the ex vivo circulation of the heparinized extracorporeal circulation catheter with the Ti, Ti − OH, and M10 samples and SEM image of thrombi (**f**) [224]

Layer-by-layer (LBL) method as a thin-layer fabrication method consisting of the deposition of alternating layers of materials by means of positive and negative species with opposite charges. Besides, the LBL method is called liquid phase epitaxy which can generate thin MOF coatings of specific thickness with good homogeneity. The different substrates such as metals, glass, and polymers are utilized in the layer-by-layer self-assembly technique, for instance titanium for bio-film inhibition [208, 209]. The mentioned method is indicated to supply crystalline, uniform, monolithic, and MOF thin films with high orientation on functionalized substrates [210]. In addition, this coating method is beneficial for controlling MOF structural inter-penetration and is applied to fabricate membranes [211], drug delivery [212] and sensors [213]. The layer thickness of MOF can be changed by selecting the appropriate number of growth cycles from nanometers to micrometers in the LBL method [214]. MOF thin films synthesized using the LBL method are commonly called surface-mounted metal–organic frameworks (SUR-MOFs) by step-by-step deposition process. The layer-by-layer method is usually performed at room temperature or low temperature. The reactants are separated and the film is grown by repeated immersion in two solutions containing organic linker and a metal source (Fig. 10a and b). Different materials such as nanoparticles, polymers, proteins, and DNA can be used to deposit multilayers from their solutions or suspensions which also allows to further extend the number of compounds by changing the organic ligands or metal during fabrication, yielding multi-heterolayers [215]. Relatively good MOF loading into substrates containing the carboxylic functional or pyridyl group is usually considered to help enhance MOF nucleation [216]. To improve this method, it can be combined with atomic layer deposition method as a MOF nucleation layer to improve MOF growth on various materials [217]. Besides, MOF coatings can be used as anti-bacterial agents due to their structure, composition, and high surface volume, which is the advantage of MOF surface coatings as a novel high-performance material with anti-bacterial capabilities [218]. Li et al. synthesized stimulus-controlled LBL process by electrochemical-coupling layer-by-layer method in which the dimerization of N-alkyl-carbazole by an electrochemical stimulus from an electrode surface [219]. The usage of surface treatments in order to control the growth of the MOF catalysts is highly advantageous. Research has focused on MOF growth from rigid substrates like gold, silica, and alumina [220, 221]. The immobilization of Cu-based MOFs (CuBTC) on a cotton substrate is synthesized by LBL method [222]. The reversible photochromic molecule azobenzene was fabricated into pores of MOFs using a modified liquid-phase epitaxial layer-by-layer approach [223]. Under UV and visible light irradiation, the thin film has effective guest encapsulation and isomerization between trans- and cis-azobenzene in MOF pores. This material is capable as a heterogeneous catalyst for the generation of NO using S-nitrosocysteamine as the substrate. In another study, the coating of copper-based surface-attached MOF of Cu(II) benzene-1,3,5-tricarboxylate on alkali-activated titanium surface is prepared by the LBL method for NO generation [224]. The results indicated that the CuBTC coating (with ten cycles) demonstrated ideal NO release and improved proliferation of endothelial cells, suppressed growth of smooth muscle cells and macrophages, and prevented platelet adhesion and activation. Besides, this coating prevented thrombosis and decreased neointimal hyperplasia and the inflammatory response of titanium after implantation in vivo (Fig. 10c and d).

### Spray coating of MOFs

**Fig. 11:**
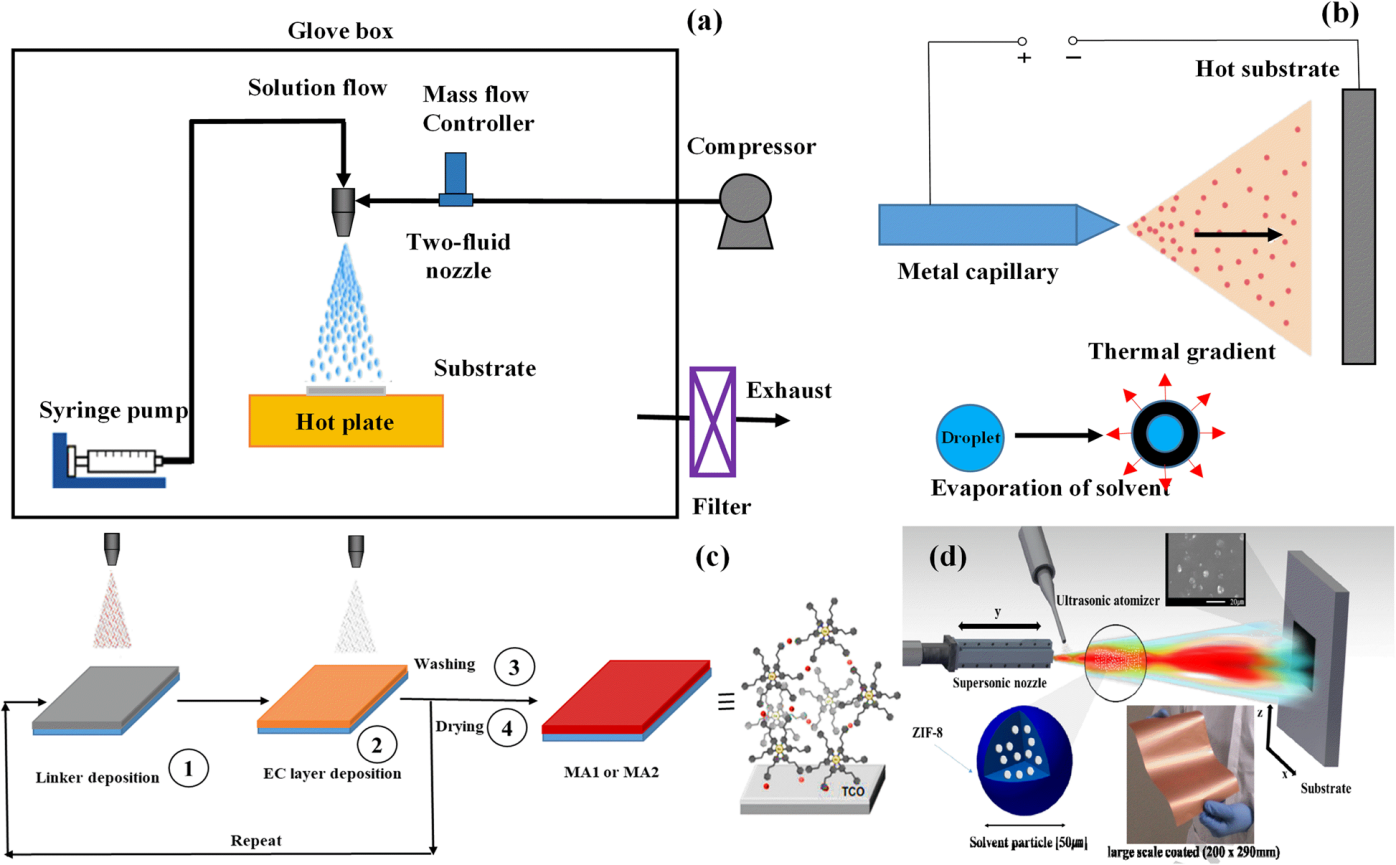
A schematic diagram for the fabrication of the MOF thin films with the spray-assisted process (**a**), schematic diagram of electrospray deposition for synthesizing ZIF-7 membranes (**b**), the fabrication of electrochromic molecular assemblies by ultrasonic spray coating (**c**), schematic of the custom-built supersonic spray-coating system (**d**) [233]

Spraying is a well-known method for producing metal and ceramic coatings. The cold spray process, like other material processing methods, has the advantage that it is a solid-state process that results in many unique coating properties. Its primary disadvantage is the plastic deformation, which leads to a loss of elasticity of the surface coating [225]. On the other hand, thermal spray coating is a general term for a set of processes that uses a plasma, electrical, or chemical combustion heat source to produce a protective layer [226]. The spray coating method is mainly used for the large-scale production of MOF powders. The sprayed droplets of the MOF precursor solution are heated by evaporation to form self-assembled metal ions and organic linkers [227, 228]. Spray self-assembly methods, compared to traditional impregnation methods, can shorten the contact time between the polyelectrolyte and the charged surface and usually do not require drying [229]. For the first time, a spray-assisted liquid-phase epitaxy process consisting of a sequential spray of a metal ion solution, an organic bonding solution, and a pure solvent for preparing directional HKUST-1 thin films has been studied [214]. In another study, HKUST-1 thin films are prepared by a spray-assisted process that involves spraying an HKUST-1 precursor solution via two-liquid nozzle on heated substrates such as Si(100) wafer, a glass plate, and porous alumina [230] (Fig. 11a). Melgar et al. synthesized a direct spraying method for fabrication of ZIF-7 MOF on the surface by the electrospray deposition [231]. This method grows from ZIF-7 membranes uniformly, well-formed and thermally stable, which grows successfully on porous alumina that ZIF-7 membranes are between 2 μm to 22 μm thick (Fig. 11b). The on-surface formation of homogeneous and uniform metalloorganic films on transparent conducting oxides (TCOs) on glass or flexible poly (ethylene terephthalate) (PET) via ultrasonic spray coating was fabricated (Fig. 11c). These MOF thin films were subsequently used into laminated electrochromic devices containing a lithium-based gel electrolyte [232]. First application of high-rate supersonic cold spray coating to deposit thin films of ZIF-8 onto glass and copper substrates was studied by Kim et al. [233]. This high-velocity deposition approach with a particle velocity of ~ 500 m·s-1 was able to fabricate a textured crystalline film (Fig. 11d). In another study, the diamine-appended MOF composites were deposited on the surface through facile spray coating and used for effective indoor carbon dioxide capture [234]. Compared with the gel typical materials, SBA-15 indicates special properties like uniform pore size, hexagonal, and cylindrical channels and large surface area. For example, Oliveira group’s reported that it synthesized a Cu-BTC MOF on mesoporous SBA-15 silica substrate by using a new approach of the layer-by-layer spray method [235]. The thin film polymer using a MOF-templating system which contained porphyrin molecules, was synthesized in a LBL method by subsequent spray coating of a substrate. Also the results showed high anti-bacterial activity against pathogens through visible light-promoted generation of reactive oxygen species [236]. However, a variety of thermal injection technologies have been developed such as flame injection, gun spray, high-speed (oxygen or air or liquid) fuel, plasma spray, and plasma transferred arc spraying techniques. Still, it seems that some of these methods are not suitable for the production of MOFs. Amongst, Plasma spray coatings are a deposition technique commonly applied to enhance the osseointegrative properties of medical implants due to improvement of the microstructural, mechanical, biological and anti-bacterial properties of the coated implant [237–240]. For instance, Singh et al. deposited calcium silicate-reinforced hydroxyapatite on the Ti6Al4V substrate by using atmospheric plasma spray to progress the corrosion resistance and bioactivity [34].

### Gas phase deposition of MOFs

**Fig. 12:**
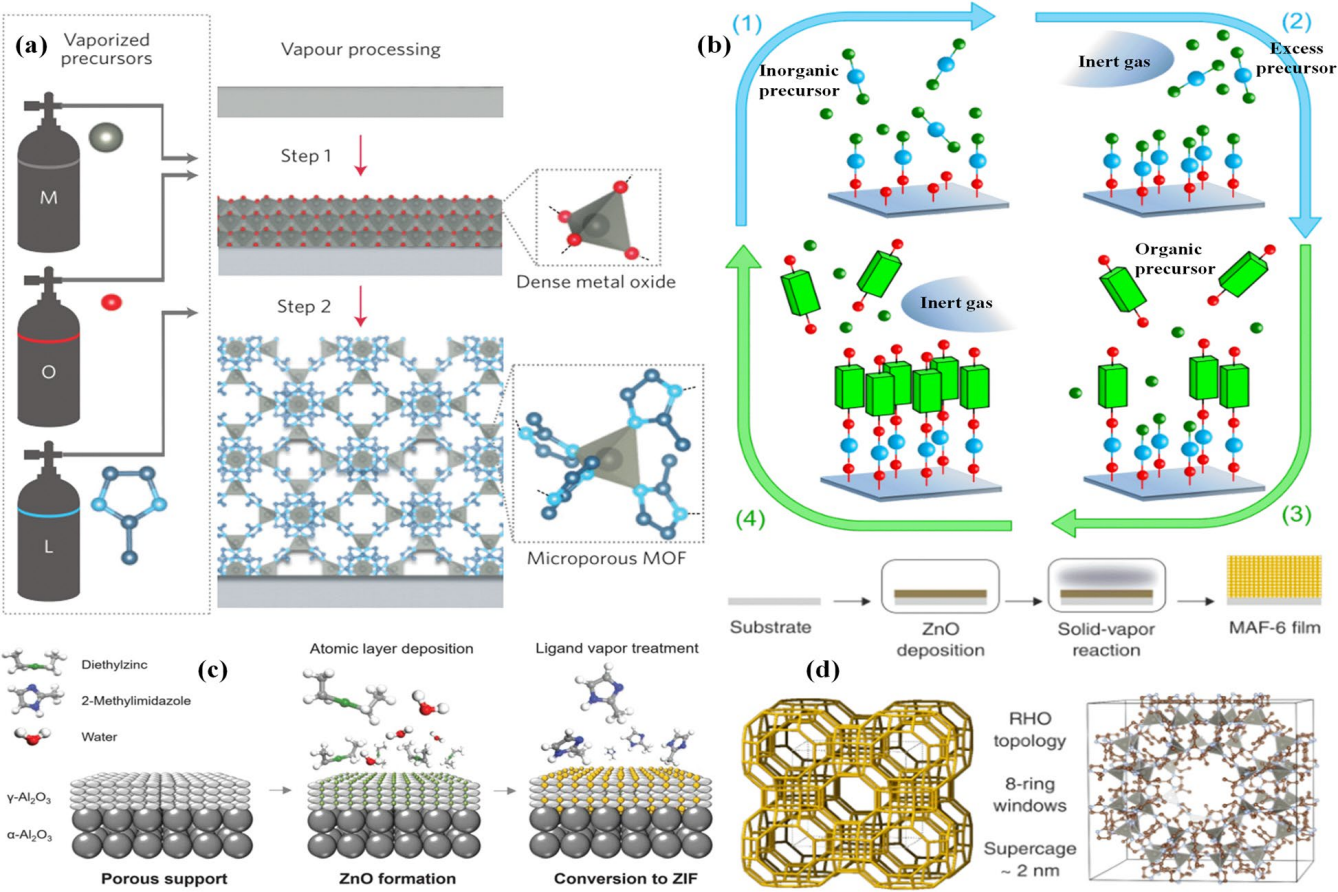
Chemical vapor deposition of ZIF-8 thin films (**a**) [244], schematic illustration of the ALD/MLD based MOF thin film growth (**b**) [247], schematic of the vaporphase ligand-induced permselectivation membrane fabrication process (**c**) [249], schematic representation of the film CVD and its topology (**d**) [250]

Developing gas-phase MOF thin film growth methods is beneficial since such procedures can broaden the use of MOFs for different medical, chemical and electronic applications. The development of solvent-free and gas-phase growth methods of MOF thin films was considered by researchers due to the limitations of the solvent-based MOF film growth methods, including contamination and corrosion of the manufacturing system as well as additional costs for safe disposal [241]. In addition, the MOF films can be fabricated as either crystalline or amorphous materials [242]. Highly crystalline copper(II)terephthalate (Cu-TPA) MOF on p-type Si (100) substrates at 180–280 ℃ by MLD approach by using Cu(thd)_2_ (copper 2,2,6,6-tetramethyl-3,5-heptanedione) and terephthalic acid (TPA) as precursors was synthesized by Ahvenniemi et al. [243]. In another study, the gas-phase ZIF-8 thin film growth method by depositing 2-methylimidazole at 100 °C were synthesized using chemical vapor deposition on ZnO layers grown by atomic layer deposition [244] (Fig. 12a). The steam-assisted chemical vapor deposition method was used to directly fabricate highly crystalline ZIF-67 thin films at the temperature < 125 °C [245]. The ZIF-67 chemiresistors show responses to the gas molecules, which can diffuse into the cage of ZIF-67 at room temperature. In another work, the gas-phase MOF growth adopting physical vapor deposition of Cu and chemical vapor deposition of H_3_BTC to grow a HKUST-1 thin film under vacuum was studied [246]. Atomic layer deposition (ALD)/ molecular layer deposition (MLD) can be used to synthesize high-quality thin films and coatings with thickness and subsequently composition control on the molecular scale. These processes are used to prepare new multilayer and nanostructure architectures by the combination of various inorganic, organic and hybrid materials. The hybrid inorganic–organic films include one inorganic and one organic precursor, and the ALD/MLD cycle can be separated into four stages consisting of precursor pulsing and intermediate purging steps [247] (Fig. 12b). In general, these MOFs were prepared under the atmosphere of inert gas (Ar or N_2_) or vacuum [248]. The liquid/gel-free ligand-induced permselectivation (LIPS) approach to synthesize high-performance ZIF-8 membranes for C_3_H_6_/C_3_H_8_ separations was reported by Ma et al. [249]. The atomic layer deposition was applied to fabricate zinc oxide (ZnO) deposits on top and inside the g-alumina layer, and subsequently the ZnO layer was transformed into a selective and permeable ZIF-8 barrier under 2-methylimidazole ligand vapor environment (Fig. 12c). The large-pore MOF MAF-6 based on the reaction of ZnO with 2-ethylimidazole vapor at temperatures ≤ 100 °C was fabricated [250]. The porosity of these MOF-CVD films and the size of MAF-6 supercages are 2 nm in diameter (Fig. 12d).

### Electrochemical deposition of MOFs

The advantages of the electrochemical technique for MOF coatings, include good quality coatings at low temperatures, with short reaction times, saving energy, and without specialized equipment compared to the traditional coating process. The limitation of this technique is that the growth substrates must be conductive [142, 173]. In general, three methods, including anodic deposition and electrophoretic deposition, and cathodic deposition have been used to fabricate MOF thin films by electrochemical methods. In the anodic deposition process, a metal electrode is applied by electrochemical dissolution of the metal anodes using high positive voltages. Then the electrically fabricated metal ions react easily with the organic bonds in the electrolyte. Finally, thin layers of MOF grow on the anode [251]. In the electrophoretic deposition, two conductive electrodes are immersed in solutions containing superficially charged MOF particles. After applying voltage between the two electrodes, the electric field generated directs the MOF particles towards the electrode with the opposite charge, resulting in the formation of thin MOF layers [252]. In cathodic deposition of MOF thin films, inert electrodes (working electrode and counter electrode) are used as inert chemical separators and they are used as the source of electrons without participating in the reactions that make up the MOF. A key stage in cathodic deposition is to obtain a local alkaline region near the cathode where the organic ligands are protonated [253]. The electrochemical deposition of MOF thin films and their various applications were compared with other works and summarized in Table 1.

**Table 1:** Summary of electrochemical deposition of MOF thin films and their various applications

MOF	Substrate	Electrodeposition Method	Solvent	Electrolyte	Application	Ref
Cu -based MOF (MOF-199)	Nickel foam	Anodic	DMF	tributylmethylammonium methyl sulfate (MTBS)	Hydrogen production	[254]
Cu-BTC (MOF-199)	Copper plate	Anodic	H_2_O/ethanol	[C8mim][Cl], [C12mim][Cl]	Detection of ethanol and methanol vapors	[255]
Cu_3_(BTC)_2_	Copper plate	Anodic	H_2_O/ethanol	NaBH_4_	reduction of p-nitrophenol to p-aminophenol	[256]
Cu -based MOF	Copper plate	Anodic	H_2_O/ethanol	phosphotungstic acid (PTA)	Detection of bromate	[257]
Zn-BTC	Zinc plate	Anodic	Ethanol	1,3,5-benzenetricarboxylic acid	Detection of nitro explosives	[258]
HKUST-1	Copper plate	Anodic	H_2_O/ethanol	1-methyl-3-octylimidazolium chloride	humidity measurement	[259]
HKUST-1	Copper plate	Anodic	water	H2SO4, phosphate buffer 6 (PBS6) and NaOH solutions	Oxygen reduction	[260]
HKUST-1	Cu rod	Anodic	water	-	Catalytic hydrolysis of carbonyl sulfide	[261]
NiBTC	Ni foam	Anodic	Ethanol	HCl solution	Hydrogen Evolution	[262]
MIL-53(Al)	Al plate	Anodic	-	-	Conversion of methane to methanol	[263]
MOF-5	Zn tablet	Anodic	-	-	hydrogen evolution reaction	[264]
Gd-BTC	Gd foil	Anodic	-	-	Detection of 2,4-dinitrotoluene	[265]
Cu-BTEC	Poly ortho aminopheno	Cathodic	DMF	Tetrabutylammonium tetrafluroborat (TBTA))	Capacitors	[266]
Cu-TDPAT @CCQDs	Nickel foil	Cathodic	-	-	Determine tyrosine	[267]
Fe/Ni-BTC	Nickel foam	Cathodic	DMF	KOH	Oxygen evolution	[268]
Ni-BDC-NH_3_ /CCQDs	Copper foam	Cathodic	DMF	2-aminoter-ephthalic acid	Detection of penicillamine	[269]
Zn-BTC	Zinc plate	Electrophoretic	DMF	ionic liquids	Reduction of CO_2_ to CH_4_	[270]
Ln@UiO-66	FTO	Electrophoretic	H_2_O/ethanol	CH_2_Cl_2_	Detection of temperature	[271]
UiO-66/C-QDs	Zinc plates	Electrophoretic	Water, ethanol, and acetone	CH_2_Cl_2_ solution	Detection of temperature	[272]
MIL-100 (Fe)	platinum foil	Electrophoretic	Water	Li2SO4, Na2SO4, K2SO4, Cs2SO4, MgSO4, potassium acetate, and KNO3	Supercapacitor	[273]

### Others methods

The depositing of MOF nanoparticles on substrates can be performed by other methods. For instance, the growth of MOF thin films based on adapting the liquid-phase epitaxy approach to the spin-coating method has been developed [64]. The applicability of this new coating/LPE method for MOF thin films varies in thickness from one micrometer to a nanometer scale. The seeded nucleation mechanism for the growth of MOF-5 can be performed directly on solid substrates [274]. The electrostatic LPE-Layer-by-Layer assembly approach offers several benefits including versatility, control over coating thickness and composition, encapsulation of sensitive drugs or biomolecules, easy incorporation of multiple functional components, surface modification for enhanced biocompatibility, controlled drug release and scalability and reproducibility. The ceramic desert rose microparticles enable the preparation of MOF-5 in a one-pot synthesis, reducing the crystal formation time and can be used to seed MOF-5 growth on any substrate, without the need for chemical functionalization of the surface. The folic-acid-modified HKUST-1 (MOF-FA) MOFs and mixed the powder into a polycaprolactone (PCL) solution to obtain a mixed-matrix coating (PCL-MOF) and dropped alone on the surface of the AZ31 Mg alloy. The corrosion resistance of the Mg-PCL-MOF was significantly enhanced [133]. Reynolds’s group used a copper-based (CuBTTri) MOF dispersed in a polymer solution to coat medical circulation tubing via a custom-designed coating system [275]. The results indicate that these surface coatings control bacterial adhesion and found reductions in S. aureus adhesion higher than 50%.

## Conclusion and future perspectives

In the present review, we especially focus on MOF coatings for implanting biomaterials and medical applications in drug delivery and also for implant coating. Overall, implant production often involves integrating material selection processes, the design and fabrication of implants, and surface modifications via material coatings, especially MOF materials. MOF coatings has a high porosity morphology, micro and meso-pores structure, the high specific surface area and easy diffusion of reagents for drug release after deconstruction of some MOFs in the surface of implants. These materials are of growing interest due to their properties and ability to supply appropriate chemical and mechanical functions for the preparation of tissue reconstruction after design and fabrication of implants in the body. There are various challenges including faster or slower than expected degeneration of the chemical and mechanical properties of metal-based implants during the tissue remodeling process. MOF surface coatings as a high-performance method are suggested as an alternative to achieve the extended mechanical integrity of these materials. Density, thickness, solubility and mineralization capacity of MOF coatings should be considered comprehensively. Currently, various MOFs are successfully applied for biomedical and pharmaceutical applications like fixation, sutures, interference screws, fixation plates, and pins, and for dental and bone implants, meniscal and cellular guidance tubes. It is applicable to provide coatings with low porosity, strong adhesion strength, appropriate crystallinity, high chemical and phase stability by using higher corrosion resistance and biocompatible coating of modified metal alloy implants. Overall, the cells like hydrophilic surfaces but the superhydrophobic surface is not favorable to cell adhesion, proliferation, differentiation and osteogenesis. Thus, the surface properties should be changed from during implantation to reach the synergistic effect of anti-bacterial and osteogenesis. While the solubility of the coating, which is closely related to its chemical composition, mechanical and microscopic morphology will affect the degradation rate. The dense coating structure and high coating thickness will efficiently hinder the penetration of corrosion fluid, thus providing impressive initial protection for some alloys like Mg substrate for the long term. Therefore, the structural design of MOF coatings on various alloys with changeable properties can be a research hotspot in the future.

## Data Availability

All data generated or analyzed during this study are included in this published article.

## Abbreviations

MOFs: Metal–organic frameworks
Bio-MOFs: Biological metal–organic frameworks
SURMOFs: Surface-mounted MOFs
SEM: Scanning electron microscopy
UV: Ultraviolet light
NIR: Near-infrared
QD: Quantum dot
DOX: Doxorubicin
Nd: Neodymium
Ti: Titanium
GO: Graphene oxide
LDH: Layered double hydroxides
LBL: Layer-by-layer
DMF: Dimethylformamide
DMSO: Dimethyl sulfoxide
PLLA: Poly (L-lactic acid)
Mg alloys: Magnesium alloys
ZnG: Zinc gluconate
ZIF: Zeolite-imidazole
HMSN-BTA@ZIF-8: Hollow mesoporous silica nanoparticle-benzotriazole- ZIF-8
TCOs: Transparent conducting oxides
PET: Poly(ethylene terephthalate)
PLLA: Poly (L-lactic acid)
PDA: Polydopamine
LPG: Long-period grating
PEEK: Polyetheretherketone
DEX: Dexamethasone
